# Fexofenadine protects against osteoarthritis by targeting Smad2 and STAT1 to enhance anabolism and binding cPLA2 to inhibit catabolism

**DOI:** 10.1038/s41420-025-02754-9

**Published:** 2025-10-21

**Authors:** Kaiwen Liu, Xiaodi Zhang, Bin Ning, Ronghan Liu, Cheng Wang, Lei Cheng, Weiwei Zheng, Jianlu Wei

**Affiliations:** 1 Department of Orthopedic, Qilu Hospital of Shandong University, Jinan, China; 2 Department of Orthopaedic Surgery, The Affiliated Hospital of Qingdao University, Qingdao, China; 3 Central Hospital Affiliated to Shandong First Medical University, Shandong First Medical University & Shandong Academy of Medical Sciences, Jinan, China

**Keywords:** Target identification, Diseases

## Abstract

Cartilage metabolism balance chaos is crucial in the development and progression of osteoarthritis (OA), with chronic low-grade inflammation being the primary factor that leads to chondrocyte metabolic dysregulation. Fexofenadine (FFD) is a widely used commercially available anti-allergy compound, which has been shown to reduce inflammation. The present study finds FFD’s therapeutic effects in primary human ex-vivo cultures and surgically induced murine models. Mechanism study illustrates FFD exhibits chondroprotective effect through anti-nuclear factor kappa-light-chain-enhancer of activated B cells (NF-κB)-mediated inflammation and pro-Transforming Growth Factor Beta (TGFβ)-associated anabolism. Specifically, FFD directly binds to cytosolic phospholipase A2 (cPLA2), down-regulating downstream NF-κB activation, resulting in alleviated catabolism. Notably, Signal Transducer and Activator of Transcription 1 (STAT1) is first identified as FFD’s target by Drug affinity responsive target stability which shows the Gln-314 site is required. FFD blocks STAT1 binds to TGF-β type I receptor, leading to secondary SMAD Family Member 2 (Smad2) phorsphorylation, slightly enhances chondrocyte proliferation and matrix production. Importantly, further study demonstrates FFD directly binds Smad2 by the target proteins fishing technique, remarkably active TGFβ-related biological process. These findings provide new insights into the chondroprotective role of FFD with novel target and downstream pathway, offering promising avenues for the treatment of OA.

**Figure:**
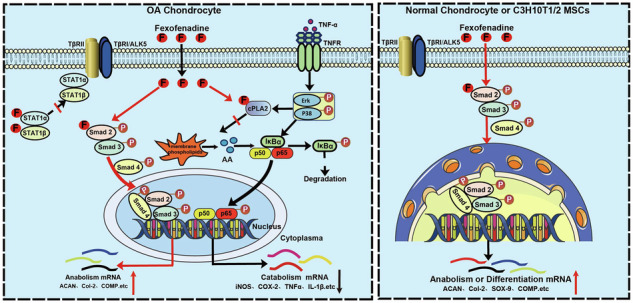
Mechanism of Fexofenadine in treating osteoarthritis. Created with BioRender.com.

Mechanism of Fexofenadine in treating osteoarthritis. Created with BioRender.com.

## Introduction

Osteoarthritis (OA) is a prevalent degenerative disease characterised by complex pathogenesis and influenced by multiple risk factors such as age, gender, and obesity. As the most common form of arthritis, OA affects approximately 7.6% of the global population (around 595 million people) [1, 2]. It is a major cause of disability in older adults and imposes a considerable economic burden. Hallmark features of OA include cartilage destruction, subchondral bone remodelling, osteophyte formation, and synovial inflammation. The progressive degeneration of articular cartilage serves as the primary hallmark of the disease [3]. Articular cartilage is primarily composed of chondrocytes and extracellular matrix (ECM) components, with chondrocytes acting as central regulators of cartilage metabolism [4]. Dysregulation of the balance between chondrocyte anabolism and catabolism is a leading cause of cartilage degradation [5, 6]. OA development involves increased catabolism driven by inflammation and decreased matrix anabolism. Despite the significant disability and morbidity associated with OA, safe therapeutic options to slow its progression remain limited. Existing non-surgical treatments primarily focus on pain management [7] but fail to halt disease progression and often come with significant side effects [8]. Thus, patients with advanced OA often undergo total joint replacement [9]. The identification of novel therapeutic agents for OA remains a critical unmet need [9], and recent advances in target discovery have contributed to a deeper understanding of OA pathogenesis.

The nuclear factor-kappa B (NF-κB) signalling pathway plays a key role in OA development and is recognised as the primary inflammatory pathway involved in the condition [10]. Elevated levels of pro-inflammatory cytokines, such as interleukin-1β (IL-1β) and tumour necrosis factor α (TNF-α), are present in the synovial fluid, synovium, subchondral bone, and articular cartilage of patients with OA, and these factors are primary activators of the NF-κB signalling pathway [11–13]. This cytokine-induced signalling pathway disrupts joint homeostasis by promoting chondrocyte catabolism and simultaneously suppressing ECM biosynthesis [14]. Therefore, inhibition of NF-κB signalling is considered a promising therapeutic strategy. Intra-articular delivery of small interfering RNA (siRNA) targeting the p65 subunit of NF-κB reduced disease severity in surgically induced OA in rats, confirming the key role of this pathway [15]. Compounds such as progranulin, Atsttrin, and 14-3-3 epsilon have demonstrated therapeutic efficacy in OA models through their effects on this pathway, supporting its translational relevance [16, 17].

The transforming growth factor-beta (TGF-β) superfamily plays a crucial role in regulating chondrocyte differentiation and maintaining metabolic balance [18]. TGF-β activates Smad2/3 signalling, promoting expression of Aggrecan (ACAN), Collagen II (COL2A1), and SRY-box transcription factor 9 (SOX9), facilitating ECM production and maintaining the chondrocyte phenotype and tissue integrity [19, 20]. However, TGF-β can also adversely affect chondrocyte metabolism via the Smad1/5/9 signalling pathway, which significantly limits the development of therapeutic strategies based on the TGF-β signalling pathway. A precise balance between Smad2/3 and Smad1/5/9 activity is required for maintaining chondrocyte homeostasis. Inhibiting TGF-β-induced phosphorylation of Smad1/5/9 while preserving Smad2/3 signalling is considered a viable therapeutic strategy for OA [21]. Thus, selective activation of the Smad2/3 axis may represent an effective approach for OA management.

Fexofenadine (FFD), a widely used and commercially available H1 receptor antagonist, is primarily prescribed for allergic rhinitis [22]. Recent research suggests that FFD reduces inflammation in diseases such as rheumatoid arthritis, intervertebral disc degeneration, and inflammatory bowel disease by inhibiting cytosolic phospholipase A2 (cPLA2), independent of classical H1R antagonism [23–26]. However, its effects on OA have not yet been investigated. This study aims to investigate the therapeutic potential and underlying mechanisms of FFD in OA. Using primary human samples and murine models, the anti-catabolic and pro-anabolic effects of FFD were comprehensively evaluated. In addition to inhibiting its known target cPLA2, FFD selectively activated the Smad2/3 pathway without triggering Smad1/5/9 signalling, and Smad2 and STAT1 were identified as novel targets. A comprehensive interaction analysis of FFD with these proteins was conducted. Overall, the findings indicate that Smad2 is essential and that STAT1 is negatively regulated during FFD-mediated promotion of chondrocyte anabolism and differentiation.

## Results

### FFD alleviates inflammation-mediated chondrocyte catabolism, enhances chondrogenic differentiation, and promotes chondrocyte anabolism

**Fig. 1:**
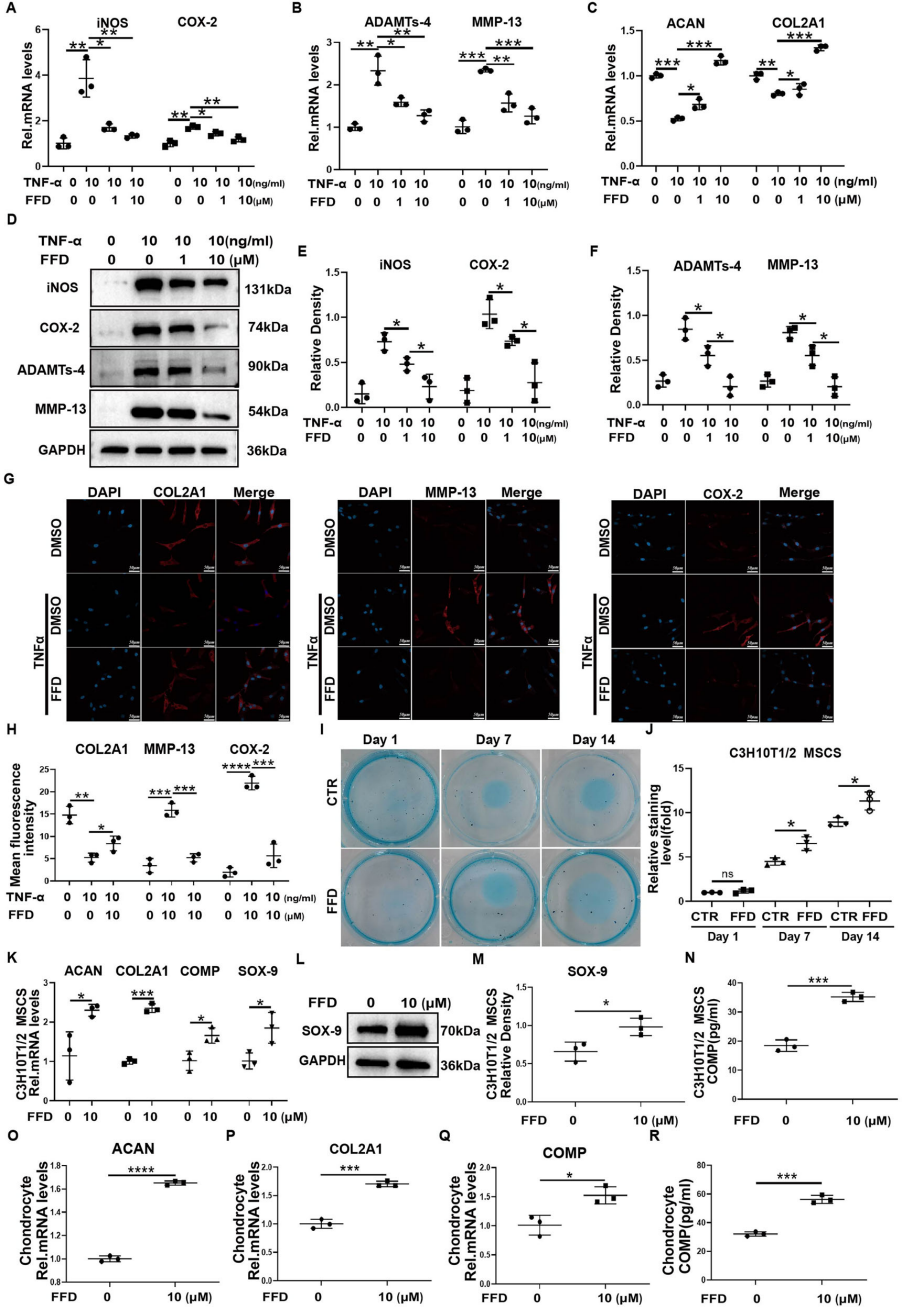
FFD alleviates inflammation-mediated chondrocyte catabolism, promotes chondrogenic differentiation, and enhances chondrocyte anabolism. **A**–**C** Human normal chondrocytes were treated with TNF-α (10 ng/mL) and FFD (10 μM) for 24 h. mRNA levels of *iNOS*, *COX-2*, *ADAMTS4*, and *MMP-13* were assessed by qRT-PCR (n = 3). **D**–**F** Human normal chondrocytes treated with TNF-α (10 ng/ml) and FFD (10 μM) for 48 h were analysed by Western blot for the corresponding proteins. Band intensities were quantified using Image J and normalised to GAPDH (n = 3). **G**, **H** Immunofluorescence staining in chondrocytes following different treatments. Quantification was performed using Image J. Scale bar = 50 μm (n = 3). **I**, **J** C3H10T1/2 mesenchymal stem cells (MSCs) were treated with or without FFD (10 μM) for 1, 7, and 14 days. Chondrogenesis was evaluated by Alcian Blue staining and quantified (n = 3). **K** mRNA expression of *ACAN*, *COL2A1*, *COMP*, and *SOX9* in C3H10T1/2 MSCs treated with or without FFD (10 μM) for 24 h (n = 3). **L**, **M** Western blot and quantification of SOX-9 protein in C3H10T1/2 MSCs after FFD treatment (10 µM) for 48 h (n = 3). **N** ELISA analysis of COMP protein levels in the culture supernatants of C3H10T1/2 MSCs treated with or without FFD (10 µM) for 48 h (n = 3). **O**, **Q** mRNA levels of *ACAN*, *COL2A1*, and *COMP* in human chondrocytes treated with FFD for 24 h (n = 3). **R** ELISA quantification of COMP in the culture supernatants of Human normal chondrocytes treated with or without FFD (10 µM) (n = 3). Statistical significance: *P < 0.05, **P < 0.01, ***P < 0.001, and ****P < 0.0001; ns not significant.

To investigate the potential of FFD to inhibit the inflammatory response in osteoarthritic chondrocytes, Human normal chondrocytes were cultured with TNF-α in the presence or absence of FFD. As illustrated in Fig. 1A, B, TNF-α significantly increased the expression of inflammatory mediators inducible nitric oxide synthase (iNOS) and cyclooxygenase-2 (COX2), along with matrix-degrading enzymes matrix metallopeptidase 13 (MMP13) and a disintegrin and metalloproteinase with thrombospondin motif 4 (ADAMTS4). FFD significantly reversed the TNF-α-induced transcriptional upregulation of these genes in a dose-dependent manner. While ECM degradation is well documented in OA, FFD treatment mitigated the TNF-α-induced downregulation of ACAN and COL2A1 expression (Fig. 1C). Furthermore, total proteins were extracted from normal chondrocytes and analysed via Western blotting, revealing that FFD dose-dependently inhibited TNF-α-induced protein expression of iNOS, COX2, MMP13, and ADAMTS4 (Fig. 1D–F). These results were further validated by immunofluorescence staining (Fig. 1G, H), which showed that FFD effectively restored the metabolic balance in TNF-α-treated chondrocytes.

To evaluate whether FFD promotes chondrocyte differentiation and anabolism, C3H10T1/2 mesenchymal stem cells (MSCs) were treated with or without FFD for 7 or 14 days. As shown in Fig. 1I, J, Alcian blue staining and subsequent quantification demonstrated that FFD significantly increased chondrogenic differentiation. The transcriptional levels of chondrogenic markers were assessed using quantitative real-time PCR (qRT-PCR) in C3H10T1/2 MSCs. As shown in Fig. 1K, the mRNA expression of *ACAN, COL2A1*, cartilage oligomeric matrix protein (*COMP*), and *SOX9* was significantly upregulated after 24 h of FFD treatment. After 48 h of incubation, total proteins were extracted to determine protein expression, revealing increased SOX9 protein levels (Fig. 1L, M). Moreover, ELISA analysis of the culture supernatants indicated elevated COMP protein levels in response to FFD treatment (Fig. 1N).

In addition to its effect on chondrogenesis, the anabolic activity of FFD in chondrocytes was further investigated by treating Human normal chondrocytes with or without FFD. As shown in Fig. 1O–Q, FFD significantly increased the transcriptional expression of ACAN, COL2A1, and COMP. ELISA results confirmed increased COMP protein levels in FFD-treated chondrocytes (Fig. 1R). These results suggest that FFD not only promotes chondrogenic differentiation but also enhances chondrocyte anabolism.

### FFD regulates cartilaginous tissue and chondrocyte metabolism ex vivo

**Fig. 2:**
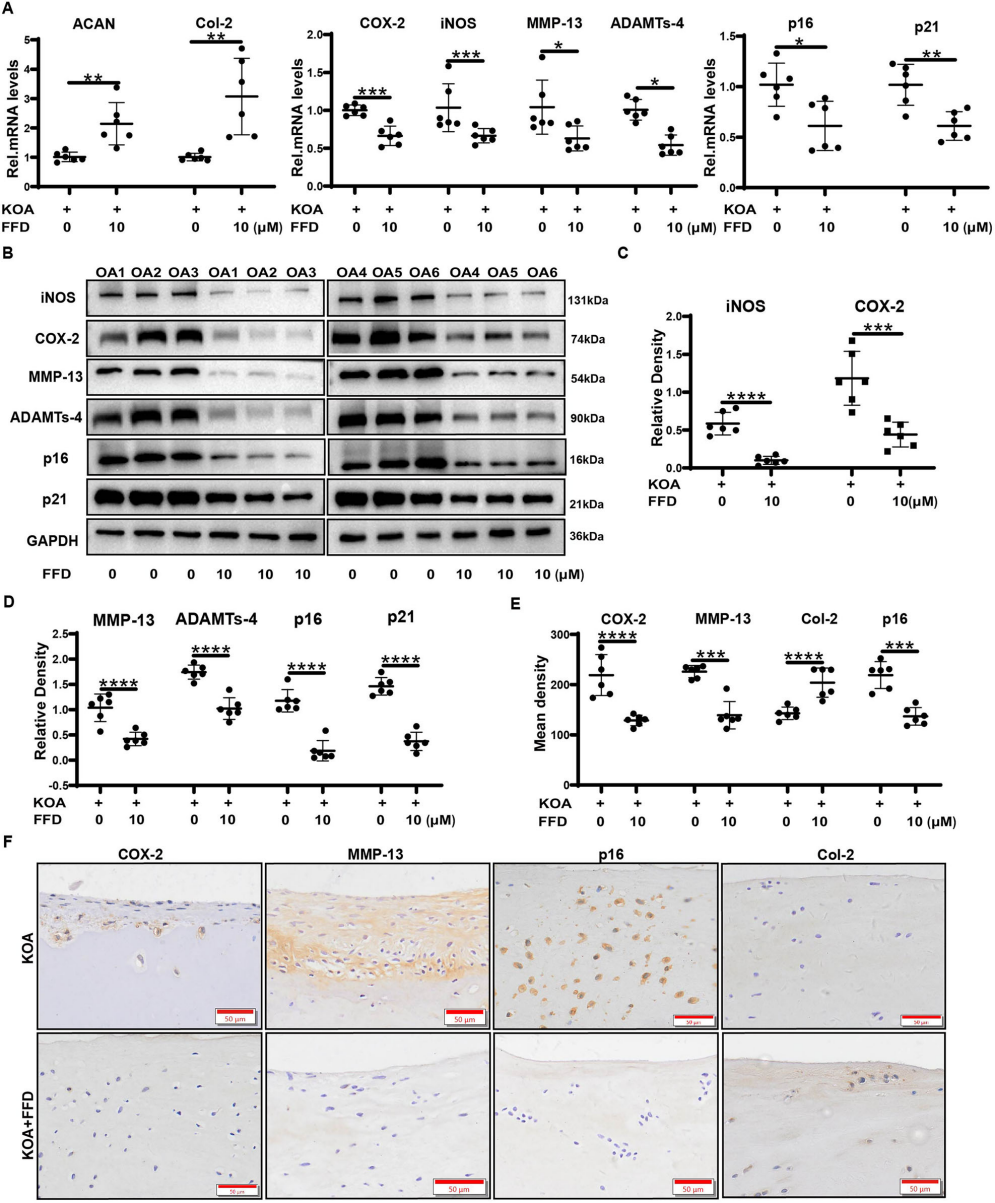
FFD regulates the metabolism of cartilaginous tissue in osteoarthritis (OA) ex vivo. Cartilage tissues from patients with OA were treated with FFD (10 µM) for 7 days. **A** qRT-PCR analysis of mRNA levels for *ACAN*, *COL2A1*, *iNOS*, *COX-2*, *ADAMTS4*, *MMP-13*, *p16*, and *p21* (n = 6). **B**–**D** Western blot analysis of protein levels of iNOS, COX-2, ADAMTS-4, MMP-13, p16, and p21, with protein bands quantification *via* Image J (n = 6). **E**, **F** Cartilage tissues were analysed *via* immunohistochemical staining for COX-2, MMP-13, COL2A1, and p16. Positive Immunohistochemical (IHC) staining was quantified using Image J (n = 6). Scale bar = 50 µm. Significant differences are indicated as follows: *P < 0.05, **P < 0.01, and ***P < 0.001.

Chondrocytes derived from patients with OA have been reported to exhibit metabolic imbalances, characterised by enhanced catabolism, reduced anabolism, and increased expression of inflammatory factors and ageing markers [12, 27, 28]. To investigate the effects of FFD on cartilaginous tissue in OA, cartilage tissue samples were collected from OA patients for ex vivo culture. The tissues were treated with or without FFD for 7 days, after which total mRNA was extracted for qRT-PCR analysis. As illustrated in Fig. 2A, FFD treatment resulted in a significant reduction in the levels of inflammatory molecules (iNOS, COX-2), matrix-degrading enzymes (MMP-13, ADAMTS4), and ageing markers (p16, p21). On the other hand, markers associated with anabolism, such as ACAN and COL2A1, exhibited significant increases. These results were corroborated at the protein level through Western blotting, as shown in Fig. 2B–D, where FFD significantly decreased the protein expression of inflammatory, catabolic, and ageing-related markers (iNOS, COX-2, MMP-13, ADAMTS4, p16, and p21). Further examination of the cultured cartilage using immunohistochemical staining revealed that FFD significantly reduced the expression of catabolism-related molecules, including COX-2, MMP-13, and p16, while significantly increasing COL2A1 expression (Fig. 2E, F).

To confirm the cellular effects of FFD, primary chondrocytes were treated with or without FFD for 48 h, followed by immunofluorescence staining. Representative images and quantitative analyses, depicted in Fig. S1A, B, demonstrated that FFD decreased the levels of COX-2, MMP-13, and p16 while increasing COL2A1 levels. Moreover, senescence-associated β-galactosidase (SA-βGal) staining, shown in Fig. 1C, indicated that FFD treatment significantly reversed senescence in chondrocytes. These findings suggest that FFD effectively modulates the degenerative phenotype of chondrocytes in patients with OA.

### FFD prevents OA progression in surgically induced OA models

**Fig. 3:**
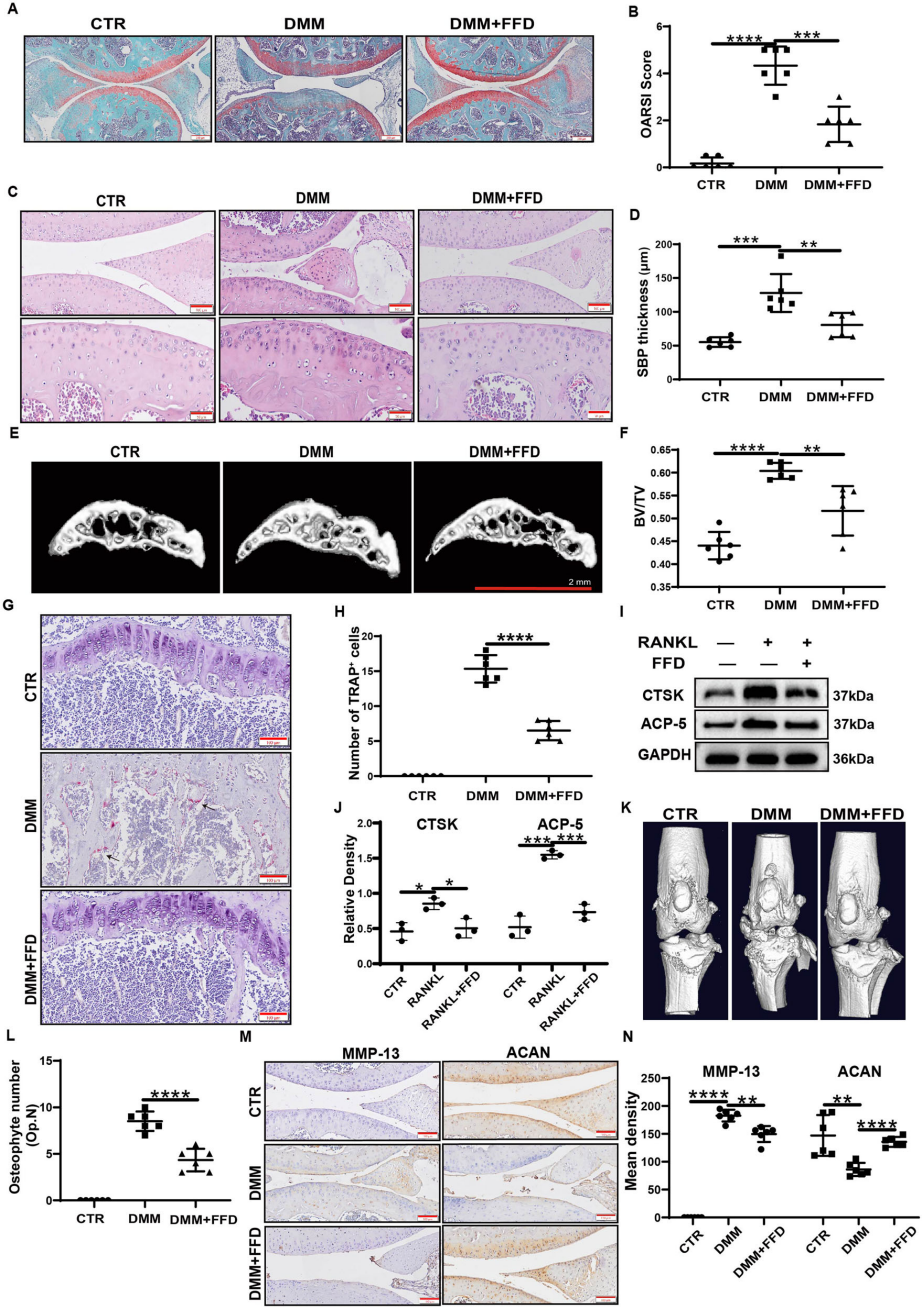
FFD prevents OA progression in surgically induced OA models. **A** Representative images of Safranin O/Fast green-stained sections of knee joints from mice treated with or without FFD for 12 weeks. Scale bar = 200 or 100 µm. **B** Quantitative analysis of the OARSI scale (n = 6). **C** H&E staining of subchondral bone in knee joint sections from mice treated with or without FFD for 12 weeks. Scale bar = 100 and 50 µm. **D** Quantification of subchondral bone plate thickness (n = 6). **E** Reconstructed micro-CT images of subchondral bone. Scale bar = 2 mm. **F** Bone volume fraction (BV/TV) analysis from micro-CT data (n = 6). **G** TRAP staining of subchondral bone. **H** Quantification of TRAP-positive cells (n = 6). **I** Western blot analysis of ACP-5 and CTSK in BMDMs treated with M-CSF (10 ng/mL) for 6 days and RANKL(100 ng/mL), with or without FFD for 7 days. **J** Band quantification of ACP-5 and CTSK based on the Western blotting assay (n = 3). **K** Representative three-dimensional micro-CT images of pathological structural changes in mouse knee joints 12 weeks after surgery. **L** Quantification of osteophyte numbers (n = 6). **M** Representative images of immunohistochemical staining of MMP-13 and ACAN in knee-joint sections from mice treated with or without FFD for 12 weeks. **N** Quantitative analysis of IHC staining using Image J (n = 6). Scale bar = 200 or 100 µm. Significant differences are indicated as follows: *P < 0.05, **P < 0.01, ***P < 0.001, and ****P < 0.0001.

To investigate the effects of FFD on OA progression, 12-week-old male C57BL/6 J mice underwent destabilisation of the medial meniscus (DMM) surgery [29]. Oral administration of FFD was initiated three days post-surgery. Tissues were collected 12 weeks after the DMM procedure was performed. As illustrated in Fig. 3A, Safranin O staining indicated significant cartilage degradation following DMM surgery. However, treatment with FFD effectively preserved proteoglycan content. Moreover, the Osteoarthritis Research Society International (OARSI) scores, shown in Fig. 3B, demonstrated that FFD treatment significantly protected against cartilage matrix loss. The characteristic loss of articular cartilage and thickening of the subchondral bone associated with OA were observed. HE staining (Fig. 3C, D) revealed that FFD effectively mitigated the reduction in subchondral bone thickness. CT three-dimensional reconstruction analysis of the subchondral bone in the tibia of mice showed that FFD significantly inhibited the bone-volume increase caused by DMM surgery (Fig. 3E, F). Previous research suggests that osteoclasts play a key role in subchondral bone remodelling during OA. To determine whether FFD-mediated protection of subchondral bone occurs through the inhibition of osteoclast formation, tartrate-resistant acid phosphatase (TRAP) staining was performed. The results indicated that FFD significantly inhibited osteoclast formation (Fig. 3G, H). To further confirm these findings, primary mouse bone marrow-derived macrophages (BMDMs) were cultured with or without FFD in the presence of RANKL (100 ng/mL) for two days, followed by Western blotting. The analysis revealed that FFD significantly reduced RANKL-induced expression of TRAP and Cathepsin K (CTSK) (Fig. 3I, J). Furthermore, micro-computed tomography (µCT) analysis revealed increased osteophyte formation in the knee joints 12 weeks after DMM surgery, which was significantly decreased by FFD treatment (Fig. 3K, L). Furthermore, immunohistochemical staining demonstrated that FFD reduced MMP-13 expression while restoring ACAN expression (Fig. 3M, N). Overall, these findings suggest that FFD protects against the development of OA in vivo.

### TGF-β and NF-κB signalling are involved in FFD-mediated cartilage homeostasis

**Fig. 4:**
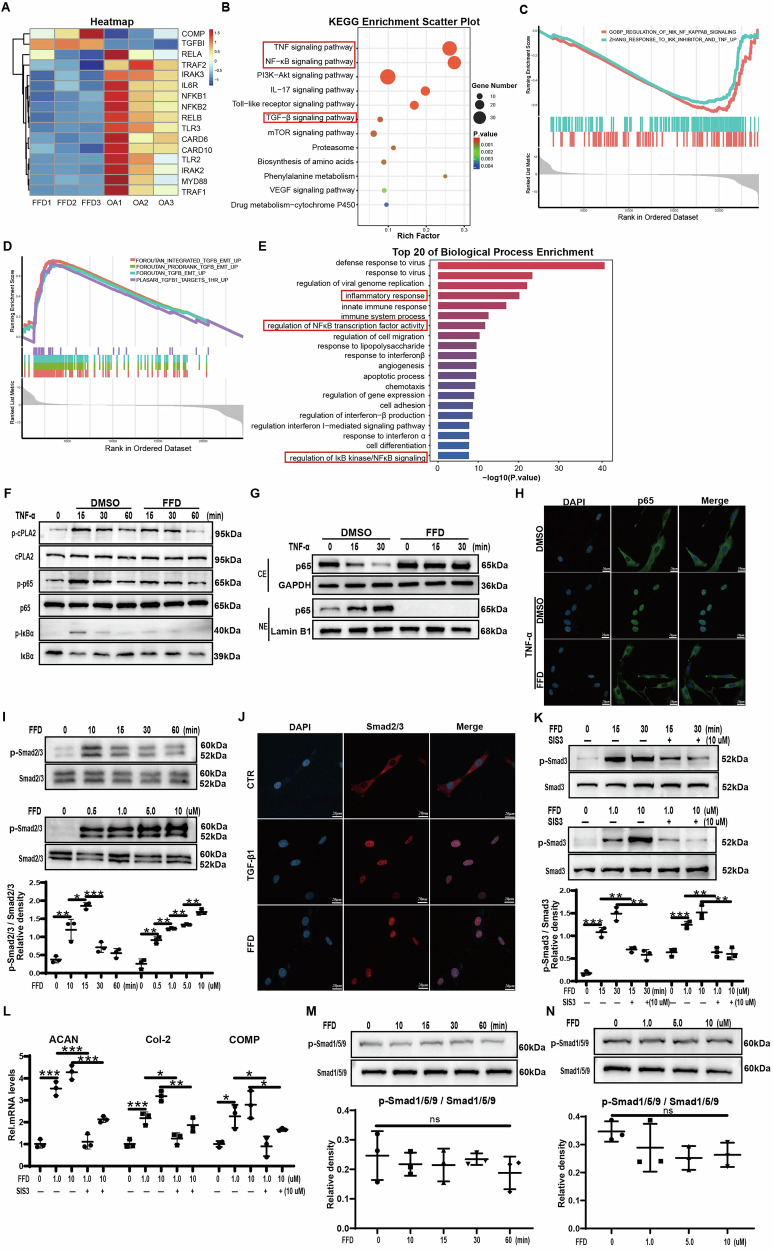
TGF-β and NF-κB signalling are involved in FFD-mediated cartilage homeostasis. **A** Heatmap showing changes in gene expression between the FFD-treated and control groups (n = 3 per group). **B**, **E** Bubble and bar graphs showing KEGG and GO enrichment results, respectively. **C**, **D** GSEA results showing downregulation of the NF-κB signalling pathway and upregulation of the TGF-β pathway after FFD treatment. **F** Western blot analysis of phosphorylated cPLA2, p65, and IκBα levels in Human normal chondrocytes. Band intensities quantified using Image J (n = 3). **G** Western blot analysis of p65 protein levels in cytoplasmic extract (CE) and nuclear extract (NE) of Human normal chondrocytes at various time points following TNF-α (10 ng/mL) with or without FFD (10 μM) treatment (n = 3). **H** Immunofluorescence staining of p65 subcellular localisation (n = 3). Scale bar = 50 µm. **I** Human normal chondrocytes were incubated with different concentrations of FFD (0.5, 1, 5, 10 μM) for 30 min or with FFD (10 μM) for different lengths of time, after which total proteins were extracted and activation of the Smad2/3 signalling pathway was assessed by Western blotting. Quantification of Western blotting results by Image J (n = 3). **J** Human normal chondrocytes were cultured for 6 h with or without FFD (10 μM). Immunofluorescence staining was used to visualise the subcellular localisation of Smad2/3. Scale bar = 50 µm, n = 3. **K** Smad3 signalling activation was assessed in chondrocytes pre-treated with SIS3 (10 μM) for 1 h, followed by incubation with FFD (10 μM) for different lengths of time or with different concentrations of FFD for 30 min. The protein expression levels of p-Smad3 and Smad3 were measured using Western blotting and quantified with Image J (n = 3). **L** mRNA levels of ACAN, COL 2, and COMP in Human normal chondrocytes pre-treated with SIS3 (10 μM) for 1 h before treatment with different concentrations of FFD for 24 h, shown by qRT-PCR (n = 3). **M**, **N** Western blot analysis of p-Smad1/5/9 and Smad1/5/9 protein levels in Human normal chondrocytes treated with FFD for different time points or different concentrations for 30 min. Significant differences are indicated as follows: *P < 0:05, **P < 0:01, and ***P < 0:001; ns not statistically significant.

To investigate the mechanisms through which FFD affects OA, cartilage samples were collected from patients, and the transcriptomes of primary chondrocytes were sequenced. Volcano plots (Fig. S2A) illustrate the changes in gene expression associated with OA, revealing that 373 genes were upregulated and 891 genes were downregulated. Several inflammation-related genes were downregulated, while multiple genes associated with anabolism displayed increased expression following FFD treatment (Fig. 4A).

To identify the signalling pathways implicated in FFD’s effect on OA progression, Kyoto Encyclopedia of Genes and Genomes (KEGG) and Gene Ontology (GO) enrichment analyses were performed. These analyses indicated that NF-κB, TNF, and TGF-β signalling pathways contributed to FFD’s effects (Fig. 4B). To further explore the downstream impacts of these pathways after FFD exposure, gene set enrichment analysis (GSEA) was conducted. As illustrated in Fig. 4C, D, FFD treatment inhibited NF-κB signalling while activating TGF-β signalling. The biological processes enriched among these genes were primarily associated with the inflammatory response, apoptosis, and regulation of NF-κB transcription factor activity, including control of IκB kinase/NF-κB signalling (Fig. 4E). These findings indicate that the protective effects of FFD are mediated by the inhibition of NF-κB signalling and upregulation of TGF-β signalling.

### FFD targets the cPLA2/NF-κB signalling pathway

To further elucidate the underlying mechanisms, phosphorylation of p38, ERK1/2, IκBα, p65, and cPLA2 was assessed over time in the presence of TNF-α. As shown in Fig. 4F and Fig. S2B–D, FFD did not affect TNF-α-induced phosphorylation of p38 or ERK1/2. However, it significantly inhibited TNF-α-stimulated phosphorylation of cPLA2 and p65, and prevented degradation of IκBα. Immunofluorescence analysis further confirmed the inhibitory effect of FFD on TNF-α-induced IκBα phosphorylation (Fig. S2E, F). As the activation of NF-κB signalling depends on nuclear translocation of the p65/p50 dimer, Western blotting and immunofluorescence assays were used to evaluate this process. The results showed that FFD significantly inhibited TNF-α-induced nuclear translocation of p65 in chondrocytes (Fig. 4G, H and Fig. S2G).

Recent investigations have identified cPLA2 as a direct molecular target of FFD binding [23]. FFD displays anti-inflammatory activity in various inflammatory settings by binding to cPLA2 and inhibiting its phosphorylation. To confirm this interaction in chondrocytes, DARTS assays were conducted, showing that cPLA2 remained undigested by the protease following incubation with FFD (Fig. S2H, I). To corroborate these findings, arachidonic acid (AA) concentrations were measured in the culture supernatants of chondrocytes by ELISA. As presented in Fig. S2J, FFD significantly reduced TNF-α-induced AA production in a dose-dependent manner. Further supplementation with AA led to increased IL-1β expression (Fig. S2K), indicating that AA could reverse the inhibitory effects of FFD on TNF-α. These findings demonstrate that FFD suppresses TNF-α-induced inflammatory catabolism in chondrocytes by targeting the cPLA2/NF-κB signalling axis.

### FFD promotes chondrocyte anabolism via the TGF-β/Smad2/3 signalling pathway

The TGF-β/Smad2/3 signalling pathway is essential for preventing ECM degradation, enhancing ECM component synthesis, and promoting cell proliferation [7, 30]. To further investigate the molecular mechanisms by which FFD promotes chondrocyte anabolism, its effect on the TGF-β/Smad2/3 signalling pathway was evaluated. Normal chondrocytes were treated with FFD at varying concentrations and time points, followed by Western blot analysis of total and phosphorylated Smad2/3. The results demonstrated that FFD induced phosphorylation of Smad2/3 in a dose- and time-dependent manner (Fig. 4I).

Given that phosphorylated Smad2/3 translocate to the nucleus to exert their biological functions, immunofluorescence staining was conducted to observe Smad2/3 localisation. As shown in Fig. 4J, FFD treatment significantly increased the nuclear translocation of Smad2/3. Furthermore, the use of the Smad3 pathway inhibitor, SIS3, significantly reduced FFD-mediated phosphorylation of Smad3 (Fig. 4K). Therefore, blocking Smad3 signalling led to a significant decrease in the transcriptional levels of anabolic markers, including ACAN, COL2A1, and COMP (Fig. 4L).

In chondrocytes, activation of the Smad1/5/9 signalling pathway promotes hypertrophic chondrocyte differentiation, which is different from the activation of the Smad2/3 pathway. To verify whether FFD could activate the Smad1/5/9 signalling pathway, normal chondrocytes were treated with FFD at various concentrations and time points. The findings indicated that FFD did not phosphorylate Smad1/5/9 (Fig. 4M, N). These results suggest that the pro-anabolic effects of FFD depend on the activation of the TGF-β/Smad2/3 signalling pathway.

### STAT1 is a novel target of FFD and plays an important role in enhancing the anabolism of OA chondrocytes

Previous studies have found that FFD targets H1R in allergic rhinitis and cPLA2 in rheumatoid arthritis [22–26]. To investigate whether the anabolic effects of FFD in chondrocytes were mediated through H1R and/or cPLA2, H1R and cPLA2 were knocked down using siRNA in Human normal chondrocytes (Fig. S3A). The knockdown of either H1R or cPLA2 did not affect the FFD-mediated upregulation of ACAN and COL2A1, suggesting that neither H1R nor cPLA2 is involved in the pro-anabolic effects of FFD on chondrocytes (Fig. S3B, C).

**Fig. 5:**
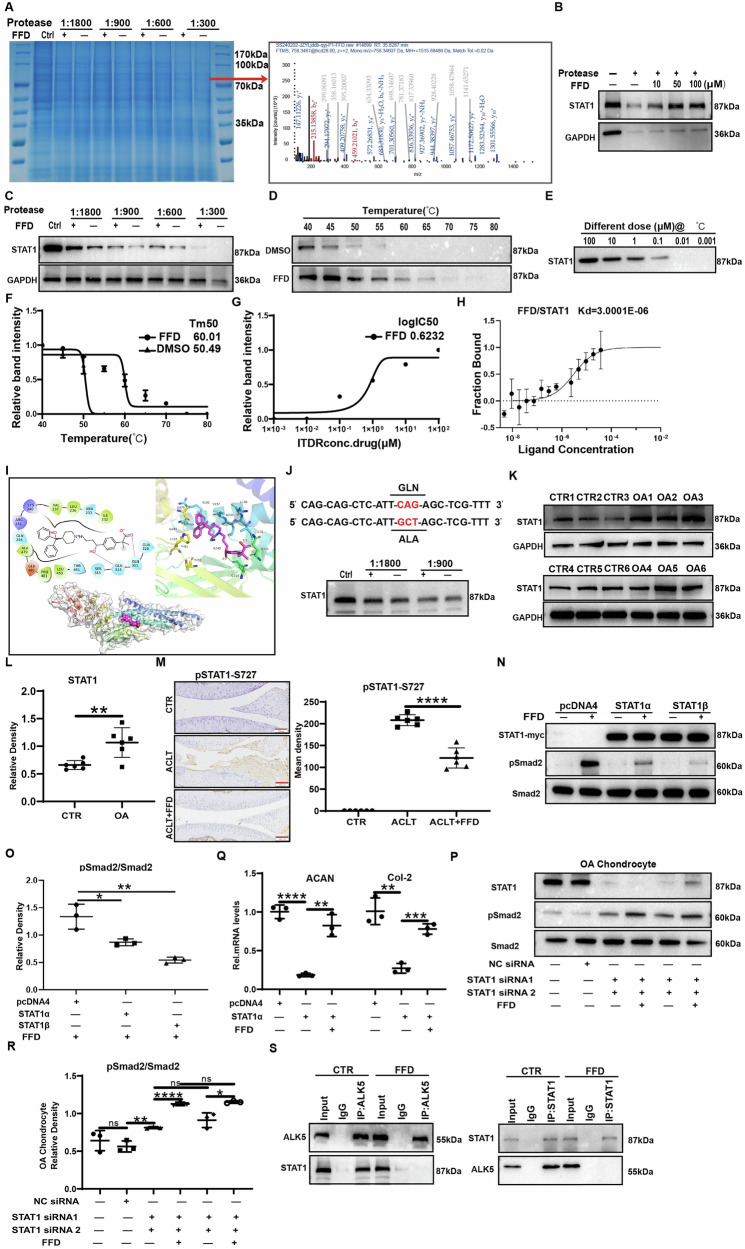
STAT1 is a novel target of FFD and mediates anabolic enhancement in OA chondrocytes. **A** Coomassie Brilliant Blue staining of SDS-PAGE gels after the DARTS experiment; bands with molecular weights around 80 kDa were protected by FFD. Mass spectrum of STAT1. **B** Western blot analysis of STAT1 protein levels in Human normal chondrocytes treated with increasing concentrations of FFD (0, 10, 50, 100 μM) under proteolytic digestion. **C** Human normal chondrocytes were digested with different concentrations of proteases with or without FFD, after which STAT1 protein levels were determined using Western blotting. **D**, **F** Human normal chondrocyte lysates were denatured at different temperatures with or without FFD, after which STAT1 protein levels were assessed by Western blotting and Image J software (n = 3). **E**, **G** Isothermal dose-response curve (n = 3). **H** MST assay showing affinity between FFD and purified His-tagged STAT1 protein. **I** Molecular docking simulation of the interaction of FFD with human STAT1. STAT1 is shown in cyan. Hydrogen bonding is indicated by green dashed lines in 3D plots, with values indicating the bonding distances. Hydrogen bonding is indicated by purple arrows in 2D plots. Hydrophobic amino acids are shown in green, acidic amino acids in red, basic amino acids in blue, and uncharged polar amino acids in cyan. **J** Human normal chondrocytes were transfected using a myc-tagged STAT1 point mutant plasmid. Western blotting was used to assess MYC signalling after the DARTS experiment. **K**, **L** STAT1 protein expression in chondrocytes from healthy individuals and OA patients shown by Western blotting, and quantified using Image J (n = 6). **M**, **N** Protein expression of p-STAT1 (S727) in the ACLT animal model was assessed by immunohistochemical staining. Scale bars = 200 µm (left panel) and 100 µm (right panel). Quantitative analysis of immunohistochemical staining using Image J (n = 6). **O**, **P** OA chondrocytes were transiently transfected with pcDNA4, STAT1, or STAT1β plasmids for 24 h, after which they were treated for 30 min with or without FFD (10 µM). Western blotting and quantification of phosphorylated Smad2 (n = 3). **Q** OA chondrocytes were transiently transfected with pcDNA4 STAT1α plasmids for 24 h, after which they were treated for 24 h with or without FFD (10 µM). mRNA levels of ACAN and COL2A1 were measured using qRT-PCR (n = 3). **R**, **S** Knockdown of STAT1 in OA chondrocytes by siRNA, after which the cells were treated for 30 min with or without FFD (10 µM). Smad2 phosphorylation was assessed by immunoblotting. Densitometric analysis of the gels by Image J (n = 3). **T** Co-immunoprecipitation (Co-IP) was used to assess STAT1 interaction with ALK5 with or without FFD treatment. Significant differences are indicated as follows: *P < 0:05, **P < 0:01, and ***P < 0:001; ns: not statistically significant.

To identify the specific FFD targets responsible for its anabolic activity, a drug affinity responsive target stability (DARTS) assay was conducted in combination with mass spectrometry [31]. After protein separation using SDS-PAGE and staining with Coomassie Brilliant Blue, a distinct band at approximately 80 kDa protected by FFD was observed (Fig. 5A). Mass spectrometry analysis indicated that STAT1 may participate in the FFD-TGF-β/Smad2/3 signalling pathway, given the relevance of TGF-β/Smad2/3 in FFD-mediated anabolism. To investigate whether STAT1 interacts with FFD, a DARTS assay was used to digest Human normal chondrocyte lysates with varying protease concentrations, followed by incubation at room temperature with FFD and subsequent Western blotting. As indicated in Fig. 5B, C, FFD effectively protected STAT1 from enzymatic digestion. To further confirm the interaction between FFD and STAT1, a cellular thermal shift assay (CETSA) was performed [32]. As demonstrated in Fig. 5D, E, FFD binding prevented STAT1 denaturation compared to the control group, particularly at 60 °C. The unchaining curves showed a robust change in the unchaining temperature (Tm) in the presence of FFD, where the Tm was 50.49 and 60.01 °C for the control and FFD-treated groups, respectively (Fig. 5F). Further, CETSA conducted at 60 °C with varying drug concentrations showed that FFD inhibited STAT1 denaturation in a dose-dependent manner (Fig. 5G). To directly evaluate the binding affinity between FFD and STAT1, the STAT1 protein was purified, and microscale thermophoresis (MST) was employed, revealing a binding strength of 3.0 μM (Fig. 5H). Molecular docking was conducted using AutoDock 4.2 software with the FFD structure and a homology-modelled human STAT1 to identify the binding sites of FFD on STAT1 [33]. The analysis indicated that FFD binds to human STAT1 mainly through hydrogen bonding, van der Waals forces, and hydrophobic interactions, with specific binding predicted at Gln314 and Gln311 (Fig. 5I). Mutation of these residues demonstrated that FFD could not bind to STAT1 when Gln314 was altered, indicating its vital role in the binding interaction (Fig. 5J).

Despite identifying STAT1 as a potential FFD-binding protein, its specific role in OA remained unclear. To clarify this, clinical samples were collected to assess STAT1 expression in cartilage tissues. Western blotting revealed elevated STAT1 protein levels in human OA cartilage (Fig. 5K, L). Moreover, phosphorylated STAT1 levels increased following anterior cruciate ligament transection (ACLT) surgery in mice, whereas FFD administration blocked this phosphorylation (Fig. 5M, N). To elucidate the role of STAT1 in the FFD-Smad2/3 axis, STAT1 overexpression was induced in OA chondrocytes using an overexpression plasmid, and the status of the TGF-β signalling pathway was examined. FFD-mediated Smad2 phosphorylation was significantly reduced in the presence of overexpressed STAT1α or STAT1β (Fig. 5O, P), leading to changes in the expression of Smad2-activated genes. Transcription levels of ACAN and COL2A1 were significantly reduced with STAT1 overexpression, while treatment with FFD restored these levels (Fig. 5Q). The loss-of-function aspect was also investigated by knocking down endogenous STAT1 expression in OA chondrocytes using STAT1-siRNA, which resulted in minimal activation of Smad2 phosphorylation. After knocking down STAT1 expression, the phosphorylation of Smad2 in OA chondrocytes was upregulated. The addition of FFD further increased Smad2 phosphorylation. (Fig. 5R, S). Previous studies have shown that in ovarian cancer cells, STAT1 can modulate the TGF-β signalling pathway by binding to the TGF-β receptor [34]. To determine whether a similar mechanism exists in OA chondrocytes, co-immunoprecipitation (Co-IP) was conducted. The results revealed that STAT1 binds to Activin Receptor-Like Kinase 5 (ALK5) in human chondrocytes, and this interaction is disrupted by FFD treatment (Fig. 5T). These findings suggest that FFD modulates the TGF-β/Smad2/3 pathway in OA chondrocytes by interfering with STAT1 binding to ALK5, therefore promoting anabolism.

### Smad2 is a novel target of FFD and required for FFD regulation of chondrocyte anabolism and chondrogenesis

**Fig. 6:**
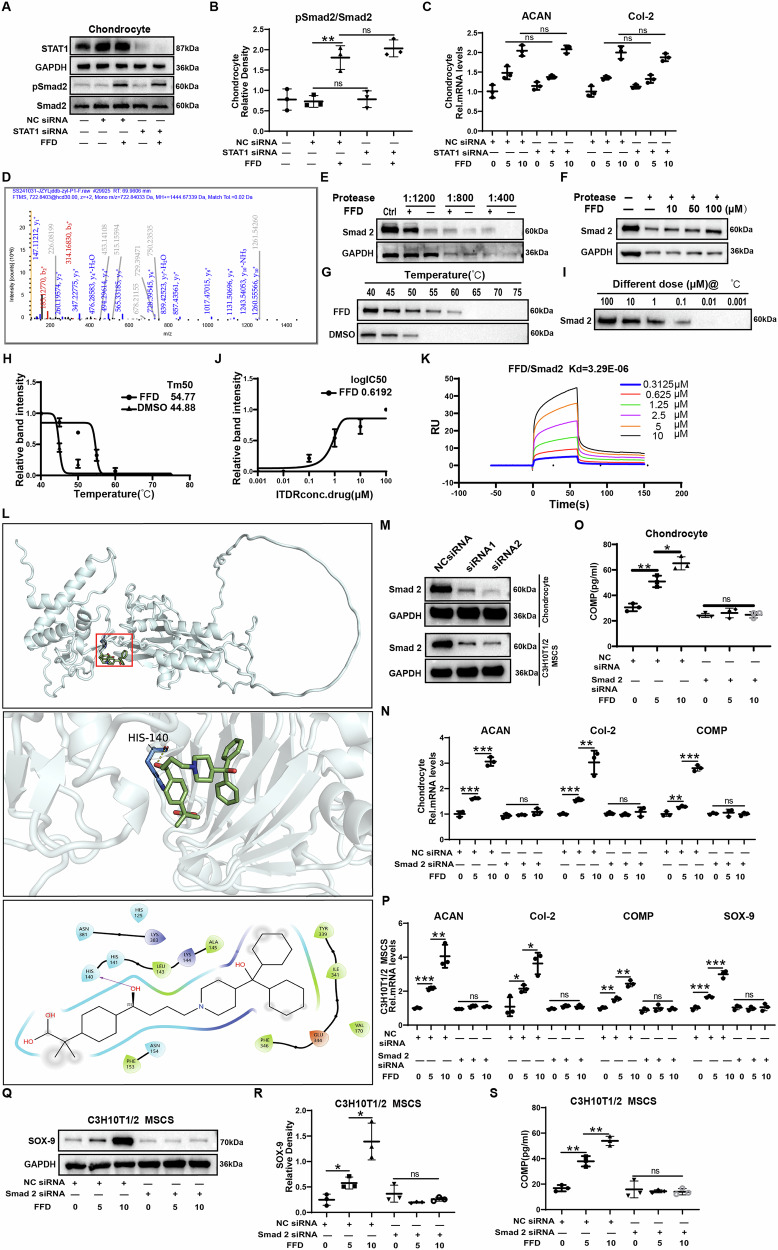
Smad2 is a novel target of FFD and is required for FFD regulation of chondrocyte anabolism and chondrogenesis. **A**, **B** *STAT1* knockdown *via* siRNA in Human normal chondrocytes, after which the cells were treated with or without (10 µM, 30 min); Smad2 phosphorylation was assessed *via* Western blot and quantified using Image J (n = 3). **C** qRT-PCR analysis of *ACAN* and *COL2A1* mRNA levels in STAT1-silenced chondrocytes treated with or without FFD (10 µM, 24 h) (n = 3). **D** Pull-down of target proteins from human chondrocytes using FFD-conjugated Fe_3_O_4_ beads, followed by mass spectrometry. **E**, **F** DARTS assay confirming FFD–Smad2 interaction. **G**, **H** CETSA demonstrating thermal stabilisation of Smad2 by FFD. **I**, **J** Isothermal dose-response assay showing Smad2 stabilisation by FFD, with Western blot and associated quantitative curve. **K** Surface plasmon resonance (SPR) assay assessing affinity between FFD and purified His-tagged Smad2 protein. **L** Molecular docking of FFD to Smad2 illustrating surface and 3D binding modes. **M** Western blot validation of siRNA-mediated Smad2 knockdown in human chondrocytes and C3H10T1/2 MSCs. **N** mRNA levels of *ACAN*, *COL2A1*, and *COMP* in Human normal chondrocytes assessed *via* qRT-PCR (n = 3). **O** COMP protein levels in Human normal chondrocyte culture supernatants measured by ELISA (n = 3). **P** qRT-PCR analysis of *ACAN*, *COL2A1*, and *COMP* mRNA levels in C3H10T1/2 MSCs (n = 3). **Q**, **R** Western blot and Image J quantification of SOX-9 protein levels in C3H10T1/2 MSCs (n = 3). **S** COMP protein levels in culture supernatants of C3H10T1/2 MSCs measured by ELISA (n = 3). Statistical significance: *P < 0:05, **P < 0:01, and ***P < 0:001; ns not statistically significant.

Degenerated chondrocytes exhibit distinct phenotypic features compared to normal chondrocytes. To further investigate whether STAT1 serves as a functional target of FFD in normal chondrocytes, STAT1 expression was knocked down in human normal chondrocytes (Fig. 6A, B). As shown in Fig. 6C, unlike the observations in degenerated chondrocytes, STAT1 knockdown in normal chondrocytes did not affect Smad2 phosphorylation. Even after STAT1 knockdown, FFD still promoted ACAN and Col2 expression in chondrocytes.

To identify the primary protein target through which FFD stimulates anabolic processes in normal chondrocytes, pull-down assays were conducted using FFD-conjugated magnetic beads incubated with lysates from these cells. Mass spectrometry identified Smad2 as a potential FFD-binding protein (Fig. 6D). DARTS and CETSA experiments subsequently confirmed the interaction between FFD and Smad2 (Fig. 6E–J). Furthermore, surface plasmon resonance (SPR) analysis using purified Smad2 protein and FFD revealed a dissociation constant (*K*_*D*_) of 3.29 × 10^−06 ^μM, indicating direct binding of FFD to Smad2 (Fig. 6K). To further characterise the FFD–Smad2 interaction, molecular docking simulations were performed (Fig. 6L). The analysis demonstrated that FFD forms a hydrogen bond with Smad2 at His140, with a bond distance of 3.1 Å. Hydrophobic contacts were identified between FFD and Leu143, Ala145, Tyr339, Ile341, Val170, Phe346, and Phe153 (green in the 2D diagram), while polar interactions were observed with His140, His141, Asn381, His125, and Asn154 (blue in the 2D diagram). These combined interactions influence the binding affinity between FFD and Smad2.

To investigate the role of Smad2 in mediating FFD-induced anabolic activity, Smad2 was effectively knocked down using siRNA (Fig. 6M). The FFD-induced upregulation of anabolic genes, including *COL2A1*, *ACAN*, and *COMP*, was abolished in Smad2-deficient chondrocytes (Fig. 6N–P). Similarly, the increased expression of stem cell differentiation markers mediated by FFD was lost following Smad2 silencing (Fig. 6Q–S). These findings demonstrate that FFD promotes anabolic metabolism and differentiation in chondrocytes through a Smad2-dependent mechanism.

## Discussion

Recent studies on OA have emphasised not only the inhibition of aberrant catabolic activity in OA chondrocytes but also the stimulation of matrix molecule synthesis and ECM remodelling in cartilage tissue [35]. FFD has been reported to exert anti-inflammatory activity in various inflammation-associated conditions by targeting cPLA2 and inhibiting the NF-κB signalling pathway [23, 26]. In this study, the role of FFD in reversing the degenerative phenotype of OA chondrocytes was systematically investigated, confirming that FFD reduces the expression of cartilage-degrading metalloproteinases and inflammatory mediators by targeting cPLA2. FFD also promoted chondrocyte differentiation and matrix molecule biosynthesis, with STAT1 and Smad2 identified as direct protein targets involved in its anabolic function. These findings offer detailed insights into the underlying mechanisms of FFD’s therapeutic action in OA. This study also provides strong support for the potential use of FFD in the treatment of other inflammation-associated disorders.

The optimal FFD concentration was first identified using an in vitro cellular model exposed to various dosages. FFD effectively promoted anabolic activity at 5 µM and 10 µM (Fig. S4A). Similarly, it was observed that FFD showed anti-inflammatory effects in a dose-dependent manner at concentrations of 0.5 µM, 1 µM, 5 µM, and 10 µM. Even at a concentration of 20 µM, FFD still displayed good anti-inflammatory effects (Fig. S4B–D). To maintain consistency, a concentration of 10 µM was used to investigate the role of FFD in regulating chondrocyte metabolic homeostasis. FFD treatment of ex vivo cultured cartilage tissues from OA patients ameliorated metabolic imbalance in both cartilage tissues and cells in vitro. Furthermore, a DMM model was established to simulate OA in mice, providing in vivo evidence that FFD could significantly prevent the development of OA. The study also demonstrated that FFD reduces pathological subchondral bone remodelling, likely through inhibition of osteoclast differentiation. Since abnormal subchondral bone turnover is a key pathological hallmark of OA and contributes to the degeneration of articular cartilage, targeting this process may represent an effective therapeutic approach [36, 37].

To elucidate the mechanisms underlying these protective effects, transcriptomic sequencing was performed on chondrocytes from OA patients. Results indicated that FFD inhibited the NF-κB pathway while simultaneously activating the TGF-β pathway. It was observed that inflammatory cytokines induce cPLA2 phosphorylation in chondrocytes, promoting membrane phospholipid hydrolysis and subsequent release of AA. AA then activates the NF-κB pathway, resulting in the release of inflammatory factors. These findings highlight the key role of the cPLA2-AA-NF-κB axis in OA-related inflammation and demonstrate that the anti-inflammatory effect of FFD is mediated by cPLA2, reducing the production of downstream inflammatory factors and matrix-degrading enzymes.

Furthermore, it was confirmed that the FFD-mediated promotion of chondrocyte anabolism was mediated by activation of the TGF-β signalling pathway, shown by the use of a specific TGF-β pathway inhibitor. TGF-β binds to its type II receptor (TβRII), recruiting type I receptors (TβRI) on the cell surface. TβRI, also known as activin receptor-like kinase (ALK), is found in two main isoforms in human chondrocytes, namely, ALK5 and ALK1. ALK5 primarily mediates the Smad 2/3 pathway, which ultimately inhibits chondrocyte terminal differentiation, while ALK1 stimulates the Smad 1/5/9 pathway, promoting growth-plate chondrocyte terminal differentiation and MMP-13 expression [38, 39]. Many recent studies have suggested that the TGF-β/Smad pathway plays a complex role in the development and progression of OA [40]. It is believed that Smad2/3 signalling is crucial for regulating cartilage homeostasis, while Smad1/5/9 signalling leads to the onset and progression of OA [20, 41]. Therefore, the maintenance of a balance between Smad2/3 and Smad1/5/9 signalling is essential for preserving chondrocyte homeostasis [21]. In the present study, we were surprised to find that FFD selectively activated Smad2/3 signalling, without affecting Smad1/5/9 signalling. This discovery clarifies the mechanism by which FFD promotes chondrocyte anabolism.

To identify the specific target through which FFD regulates OA chondrocyte anabolism via the TGF-β signalling pathway, its known targets, H1R and cPLA2, were first investigated. However, the inhibition of these known targets did not affect FFD-mediated promotion of chondrocyte anabolism, suggesting that FFD may act through entirely different, yet undiscovered targets. Through a series of experiments, including DARTS combined with mass spectrometry, CETSA, MST assays, and site-directed mutagenesis, STAT1 was identified as a novel target through which FFD promotes chondrocyte anabolism, with the STAT1 residue Gln314 being essential for the interaction with FFD. STAT1 is a member of the STAT family and is involved in various biological processes, including cell proliferation, differentiation, apoptosis, and immune surveillance [42, 43]. Recent studies have shown that STAT1 plays a role in OA. For instance, STAT1 has been implicated in inducing inflammation and damage in chondrocytes. Treatment with fludarabine can attenuate the expression of complement C3 and protect the knee joints in ageing guinea pigs by inhibiting STAT1 [44]. Furthermore, it has been found that both STAT1 and ADAMTS12 are significantly elevated in damaged cartilage from both humans and rats, as well as in chondrocytes treated with IL-1β. STAT1 can bind to the promoter region of ADAMTS12 to activate its transcription [45]. These findings align with our results, which show elevated STAT1 expression in OA cartilage. Several studies have reported interference between STAT1 and TGF-β signalling transduction. For example, STAT1 can induce the expression of inhibitory Smad7, preventing interaction between Smad3 and the TGF-β receptor and blocking TGF-β signal transduction [46]. Moreover, a physical interaction between STAT1 isoforms and TGF-β receptors in epithelial ovarian cancer, leading to functional crosstalk between the two signalling pathways, has been reported [34]. This study demonstrated that STAT1 knockdown and overexpression in OA chondrocytes provide evidence that FFD modulates the TGF-β signalling cascade through STAT1. Elevated STAT1 expression in OA chondrocytes was shown to suppress anabolic processes by binding to TβRI (ALK5), which interferes with Smad2/3 phosphorylation. On the other hand, FFD binding to STAT1 disrupts the STAT1–ALK5 interaction, which alleviates this inhibition, restores Smad2/3 phosphorylation, and increases anabolic processes.

There are gene expression differences between OA chondrocytes and normal chondrocytes, leading to distinct cell phenotypes [47]. In normal chondrocytes, STAT1 knockdown did not result in increased Smad2 phosphorylation or upregulation of anabolic genes, indicating that FFD may act through other target proteins to promote anabolic effects. Given the potential limitations of the DARTS method [48], such as protease digestion leading to the loss of some FFD-bound proteins, pull-down assays were conducted using FFD-conjugated magnetic beads to identify direct target proteins involved in FFD-mediated anabolic regulation in chondrocytes. Mass spectrometry analysis identified Smad2 as a potential binding partner of FFD. Follow-up DARTS, CETSA, and SPR experiments validated Smad2 as a target protein that interacts with FFD. Furthermore, it was shown that FFD’s regulation of chondrocyte anabolic metabolism and stem cell differentiation depends on Smad2. This also explains why FFD promotes the phosphorylation of Smad2/3 in the TGF-β signalling pathway without affecting the phosphorylation of Smad1/5/9.

The findings of this study demonstrate that FFD protects against OA through at least three pathways: (1) FFD binds directly to cPLA2, preventing downstream activation of NF-κB and consequently reducing inflammatory catabolism; (2) FFD promotes chondrocyte anabolism and differentiation by directly interacting with Smad2 and activating its phosphorylation; (3) In OA chondrocytes specifically, FFD enhances chondrocyte anabolism by binding to STAT1 and disrupting the inhibitory effect of elevated STAT1 on the TGF-β pathway (**Graphical abstract**). This study elucidated the mechanism by which FFD exerts its effects on OA, revealing cPLA2 as a target in inflammation, as well as Smad2 and STAT1 as targets involved in anabolism in osteoarthritic chondrocytes. These findings improve understanding of how FFD functions and suggest novel targets for cartilage protection in OA.

Some limitations of the study must be considered. For example, although it was shown that FFD binds directly to Smad2 and promotes its phosphorylation, the structural basis by which this interaction increases the phosphorylation susceptibility of Smad2 was not analysed in depth. Regarding FFD’s use for OA treatment, further studies are needed to determine the appropriate dosing schedules, administration methods, and treatment durations in both animal models and clinical trials in humans.

Considering that both cPLA2/NF-κB signalling and the STAT1/Smad2/3 pathway are involved in a variety of biological processes and the pathogenesis of various diseases, FFD may emerge as an innovative therapy for many diseases, significantly expanding the clinical applications of this drug.

## Materials and methods

### Animals

All animal experiments were approved by the Medical Ethics Committee of Qilu Hospital, Shandong University. All experimental procedures were conducted following approved guidelines and complied with relevant ethical regulations for animal experimentation and research. All mice (*n* = 36) were housed in a specific pathogen-free facility at Shandong University under controlled conditions (22 °C, 12-h light/dark cycle).

### Human samples

Human normal articular chondrocytes were obtained from patients with knee trauma (e.g., osteochondral fracture of the knee joint or tibial plateau fracture) undergoing arthroscopic surgery, tibial plateau fracture incision, or internal fixation procedures. Human degenerative articular chondrocytes were collected from patients undergoing surgery for knee osteoarthritis. This study was approved by the Medical Ethical Committee of Qilu Hospital of Shandong University, and informed consent was obtained from all participants.

### In vitro experiments

#### Cell culture

Human cartilage tissues were dissected into small fragments and washed three times with sterile phosphate-buffered saline (PBS). The tissue was digested in DMEM/F12 culture medium supplemented with 0.25% collagenase II (C8150; Beijing Solarbio) at 37 °C with 5% CO_2_ for 4–6 h. The resulting cell suspension was collected and seeded in six-well plates for further experiments. Surgically obtained cartilage explants were incubated with or without FFD in six-well plates under the same conditions.

The murine cell line C3H10T1/2 (CL-0325) was purchased from Procell (Wuhan, China). Murine bone marrow-derived macrophages (BMDMs) were isolated from the femurs and tibias of C57BL/6 J mice and cultured following standard protocols. Cells were seeded in six-well plates for 24 h and treated with M-CSF (10 ng/mL; PeproTech, USA). Mature BMDMs were obtained after 6 days of culture.

#### qRT-PCR

Total RNA was extracted from cells or tissues using TRIzol reagent (Thermo Fisher, USA) according to the manufacturer’s instructions. cDNA synthesis was performed using Realtime PCR Master Mix (Toyobo, Japan). SYBR Green PCR Master Mix (Toyobo, Japan) was used for amplification. Relative mRNA expression was calculated using the 2^−ΔΔCT^ method and normalised to housekeeping gene expression. Each experiment was independently repeated three times. The sequences of all primers are listed in Supplementary Table 1.

#### Western blotting

Total protein was extracted using lysis buffer (R0010; Beijing Solarbio). Protein concentration was quantified using a BCA protein quantification kit (P0010S; Beyotime). Equal amounts of protein were separated by SDS-PAGE and transferred onto PVDF membranes (1620177; Bio-Rad). Membranes were blocked with 5% non-fat milk (P0216; Beyotime) for 2 h at room temperature and incubated overnight with primary antibodies at 4 °C. After washing, membranes were incubated with appropriate secondary antibodies for 1 h at room temperature. Protein bands were visualised using the Tanon imaging system (Shanghai, China). Detailed information on the primary antibodies is provided in Supplementary Table 2.

#### Enzyme-linked immunosorbent assay (ELISA)

The concentration of COMP in culture supernatants was measured using an ELISA kit (Elabscience, Wuhan, China) according to the manufacturer’s protocol. Absorbance was read at 450 nm using a FlexStation 3 multi-mode microplate reader (Molecular Devices). Concentrations for each sample were calculated, and data were analysed using GraphPad Prism 8 software.

#### Immunofluorescence

Cells were fixed with 4% paraformaldehyde and permeabilised with 0.1% Triton X-100 for 5 min. Following blocking with 5% bovine serum albumin (BSA) at 37 °C for 1 hour, cells were incubated overnight at 4 °C with primary antibodies, then with fluorescently labelled secondary antibodies (#ZF-0316; ZSGB-BIO) for 1 h at room temperature. Nuclei were counterstained with DAPI. Fluorescence was visualised under an confocal microscope (ZEISS, LSM900), and images were taken. Detailed information on the primary antibodies is provided in Supplementary Table 2.

#### SA-β-Gal staining

Chondrocytes were fixed with 4% paraformaldehyde and stained using a SA-β-Gal assay kit (C0602; Beyotime) in accordance with the manufacturer’s instructions.

#### Nuclear translocation of Smad2/3 and NF-κB

Chondrocytes were treated overnight with FFD or TGF-β1, followed by immunofluorescence staining to assess the localisation of Smad2/3. For NF-κB translocation, chondrocytes were treated overnight with or without FFD (10 μM) and then stimulated with TNF-α (10 ng/mL) for 6 h. Immunofluorescence staining was used to detect the localisation of p65. Cytoplasmic and nuclear proteins were extracted using a nuclear and cytoplasmic protein extraction kit (P0028; Beyotime), and the expression of p65, lamin B1, and GAPDH was analysed by Western blotting.

#### Myc-STAT1 plasmid construction and transient transfection

STAT1α and STAT1β expression constructs were generated by inserting PCR-amplified cDNA into pcDNA 3.1 (Invitrogen) at *NheI* and *NotI* sites. PCR was performed using 2× Taq Master Mix (#E005-01; Novoprotein, China) with specific primers:

- STAT1α/β-F: CTGGCTAGCGCCACCATGTCTCAGTGGTACGAACTTCA
- STAT1α-R:CGAGCGGCCGCCTACAGGTCCTCCTCTGAGATCAGCTTCTGCTCTACTGTGTTCATCATACTGTCGAAT
- STAT1β-R:CGAGCGGCCGCctaCAGGTCCTCCTCTGAGATCAGCTTCTGCTCAACTTCAGACACAGAAATCAACTC)

Two constructs, STAT1α-myc and STAT1β-myc, were validated by restriction enzyme digestion and sequencing. The STAT1 mutant plasmids were constructed based on consecutive C- and N-terminal deletions of amino acid sequences relevant to functional structure. For site-directed mutagenesis, GLN-314 (CAG) in STAT1α was mutated to GCT. Primary human chondrocytes were seeded at a density of 2.5 × 10^5^ cells/well in six-well plates and transfected with 4 μg plasmid DNA (empty vector or STAT1 constructs) using Lipo8000™ reagent (C0533-0.5 mL; Beyotime).

#### Knock down of H1R, cPLA2 AND STAT1 by siRNA

Primary human chondrocytes were seeded at a density of 2.5 × 10^5^ cells/well in six-well plates and transfected with 1 μg of human siRNA targeting STAT1 (STAT1-siRNA), H1R (H1R-siRNA), cPLA2 (cPLA2-siRNA), or Smad2 (Smad2-siRNA), or a non-specific scrambled control siRNA (NC-siRNA), using Lipo8000™ transfection reagent (C0533-0.5 mL; Beyotime) according to the manufacturer’s protocol. Cells were incubated for the indicated time before further analysis.

#### Co-Immunoprecipitation (Co-IP)

Cell lysates (1 mg protein) were incubated with anti-ALK5 antibody (4 μg) at 4 °C for 4-6 h. Protein A/G Sepharose beads (sc-2003; Santa Cruz Biotechnology) were then added and incubated for 1 h at room temperature. The immunocomplexes were washed 4–6 times with lysis buffer, followed by SDS-PAGE and Western blot analysis using specific antibodies. Detailed information on the antibodies is provided in Supplementary Table 2.

### OA cartilage explants and in vivo experiment

#### DMM and ACLT animal model

Male C57BL/6 J mice (12 weeks old) were used to establish ACLT and DMM models. Anaesthesia was administered before surgery. The DMM model was constructed by transecting the anterior medial meniscotibial ligament using microsurgical scissors under a microscope. The medial meniscus was displaced with fine forceps to confirm joint instability. In the ACLT model, the anterior cruciate ligament was completely transected using a surgical blade. Sham operations were performed on the left knee of a separate group of mice. FFD (10 mg/kg) was administered orally starting on postoperative day 3. The DMM model was maintained for 12 weeks and the ACLT model for 8 weeks [17].

#### Micro-CT scanning

Knee joints from the DMM mouse model were harvested 12 weeks post-surgery, washed with PBS, and fixed in 4% paraformaldehyde for 24 h. The samples were scanned using an in vivo micro-CT imaging system specifically designed for small animals (QuantumGX2, Massachusetts, USA), with a resolution of 6 μm/pixel, a current of 88 mA, and a voltage of 90 kV. Three-dimensional reconstructions were made, and subchondral bone remodelling and osteophyte formation were assessed using 3D analysis in CT-Analyser software.

#### OA cartilage explant culture experiment

Cartilage tissue from OA patients was dissected into approximately 4 × 4 mm pieces using ophthalmic scissors. Samples were placed in 6-well plates and cultured in DMEM/F12 medium supplemented with 10% fetal bovine serum (FBS) and 1% penicillin-streptomycin. Explants were incubated at 37 °C in a 5% CO_2_ incubator for 7 days with or without FFD treatment. Total RNA and protein were extracted for RT-qPCR and Western blot analysis, respectively. For immunohistochemical analysis, explants were fixed in 4% paraformaldehyde at 4 °C for 48 h, followed by decalcification in an EDTA decalcification solution (G1105-500 mL; Servicebio) at 37 °C, with the solution replaced with fresh EDTA solution every week. After decalcification, the samples were rinsed in running water, dehydrated, embedded in paraffin, and sectioned at a thickness of 5 μm.

#### Histology staining (TRAP staining, HE staining, and S-O staining)

Knee joints from DMM mice were fixed in 4% paraformaldehyde for 24 h and followed by decalcification in EDTA decalcification solution (G1105-500 mL; Servicebio). After embedding the tissue blocks in paraffin and sectioning at 5 μm, samples were subjected to hematoxylin and eosin (H&E), tartrate-resistant acid phosphatase (TRAP), and Safranin O–fast green staining (S-O staining). Images were captured using an optical microscope (VS120-S6-W; Olympus, Japan). Cartilage matrix integrity and proteoglycan content of articular cartilage were assessed through S-O staining using the internationally recognised OARSI scoring system. Subchondral bone thickness was assessed by H&E staining, and osteoclast activity beneath the cartilage was evaluated *via* TRAP staining.

#### Immunohistochemical (IHC) staining

Paraffin-embedded sections from human OA cartilage and mouse knee joints were deparaffinised, rehydrated, and subjected to antigen retrieval using Pepsin Antigen Retrieval Solution (X1035; Beijing Solarbio). Sections were blocked with 5% BSA and incubated overnight at 4 °C with primary antibodies. After washing with PBS, sections were incubated with secondary antibodies from an immunohistochemistry detection kit (PV-9000; ZSGB-BIO) at room temperature for 1 h. Immunoreactivity was visualised using a DAB chromogenic detection kit (ZLI-9018; ZSGB-BIO), and nuclei were counterstained with hematoxylin. Images were acquired using an optical microscope (VS120-S6-W; Olympus, Japan), and staining intensity was quantified using Image J software. Detailed information on the primary antibodies is provided in Supplementary Table 2.

### RNA sequencing and transcription factors enrichment analysis

Primary chondrocytes from OA patients were extracted and treated with or without FFD (10 μM) for 24 h. RNA sequencing was performed by Lianchuan Biotechnology Co., Ltd. Differentially expressed genes (DEGs) were identified using the DEGSeq method with a threshold of *P* < 0.05 and |Fold Change | > 2. Functional annotation of DEGs was conducted via Gene Ontology (GO, http://www.geneontology.org/) and Kyoto Encyclopedia of Genes and Genomes (KEGG, https://www.kegg.jp/) analyses. GSEA was performed to identify enriched biological pathways related to DEGs. All bioinformatics analyses were conducted using the OmicStudio platform (https://www.omicstudio.cn). The RNA-seq dataset has been deposited in the NCBI BioProject database (http://www.ncbi.nlm.nih.gov/bioproject/1249411) under accession number 1249411.

### Binding of FFD to STAT1 and Smad2

#### Drug affinity responsive target stability (DARTS) and mass spectrometry

Human or rat chondrocytes were lysed using M-PER™ Mammalian Protein Extraction Reagent (#78505; Thermo Fisher). Following centrifugation, the supernatants were incubated with DMSO or FFD at room temperature for 1 h. Samples were digested with Pronase E (#HY-114158; MCE) for 15 min, and digestion was terminated with Deacetylase Inhibitor Cocktail (P1112; Beyotime) on ice. Protein samples from rat chondrocytes were resolved by SDS-PAGE and visualised with Coomassie Blue Staining (P0017F; Beyotime). Bands with a molecular weight of approximately 80 kDa, protected by FFD, were excised and subjected to mass spectrometric analysis at LC-Bio Technologies (Hangzhou, China).

#### Synthesis of FFD-modified magnetite microspheres and mass spectrometry

FFD was conjugated with carboxyl-functionalised iron oxide (Fe_3_O_4_) magnetic nanoparticles using EDC and HOBt in DMSO. The mixture was incubated in the dark on a temperature-controlled shaker for 12 h. FFD-modified microspheres were collected magnetically and dialysed in a methanol: water (1:1) solution for 48 h. The magnetite microspheres were washed thrice with deionised water. Protein complexes bound to the FFD magnetic beads were analysed by SDS-PAGE and stained with Coomassie Brilliant Blue. The gel strips were excised, and selected bands were analysed via mass spectrometry at LC-Bio Technologies.

#### Cellular thermal shift assay (CETSA)

Chondrocytes were treated with FFD (10 μM) for 1 h in a cell culture incubator. Cells were harvested, aliquoted into EP tubes, and digested at various temperatures (40–80 °C) for 3 min. Lysates were prepared by three freeze–thaw cycles and then centrifuged at 20,000 *g* to collect the supernatants. Western blotting was used to assess the stability of the target protein. For the isothermal dose-response assay, chondrocytes were treated with FFD (0.001–100 μM) at 60 °C. Subsequent steps were carried out as described above.

#### SPR experiments

The interaction between FFD and Smad2 was assessed using a Biacore T200 system (GE Healthcare, Uppsala, Sweden). Recombinant Smad2 (HY-P71536; MCE) was immobilised non-covalently on the sensor chip at a concentration of 50 µg/mL, yielding ~12,562 response units. FFD was injected at concentrations ranging from 0.3125 µM to 10 µM at 30 µL/min using 1× PBST (1.37 M NaCl, 26.8 mM KCl, 81 mM Na_2_HPO_4_, 17.6 mM KH_2_PO_4_, pH 7.2–7.4, 0.05% Tween-20) as running buffer. Binding kinetics were analysed using Biacore Evaluation Software (v1.0), fitting the binding curves in a 1:1 binding model.

#### Microscale thermophoresis (MST)

Purified recombinant STAT1 protein (RP01251; Abclonal) was labelled using the Protein Labelling Kit RED-NHS (NanoTemper, Beijing, China). FFD stock (10 mM in DMSO) was diluted in PBS. Purified STAT1 was mixed with FFD at specified concentrations and analysed using the Monolith NT.115 instrument (NanoTemper). MST measurements were performed at 40% infrared laser power and 20% light-emitting diode power. The dissociation constant (*K*_*D*_) was calculated using NanoTemper Analysis Software (v1.5.41).

#### Ligand docking

Molecular docking of FFD with human STAT1 and Smad2 (PDB structures) was performed. UCSF Chimaera was used for water molecule removal and structural preparation. AMBER14SB was employed to assign atomic charges to the protein, while online tools were used to calculate and assign amino acid pKa values and protonation states under neutral conditions. SiteMap was employed to predict the optimal binding sites. Molecular docking was performed using AutoDock 4.2. Small molecules were modelled in three dimensions using RDKit, and AM1-BCC partial charges were assigned *via* UCSF Chimaera [33, 49–51].

### Statistics and reproducibility

Statistical analyses were performed using GraphPad Prism 8.0 (GraphPad Software, La Jolla, CA, USA). Data are expressed as mean ± standard deviation (SD). For in vitro studies, each experiment was conducted independently at least three times. For animal and human cartilage explant studies, a minimum of six biological replicates were used. Comparisons between two independent groups were assessed using Student’s *t* test, while one-way analysis of variance (ANOVA) followed by Tukey’s post hoc test was used for multiple group comparisons. A two-sided *P* < 0.05 was considered statistically significant. Investigators remained blinded to group assignments, and subjects were randomised throughout the study.

## Supplementary information

Supplementary Figure

Original Western Blots

Supplementary Table. 1

Supplementary Table. 2

## Data Availability

All data associated with this study are available within the paper or the Supplementary Materials. Data are available from the corresponding author upon reasonable request.
