# Supplementary material for: Fexofenadine protects against osteoarthritis by targeting Smad2 and STAT1 to enhance anabolism and binding cPLA2 to inhibit catabolism
Source: Cell Death Discov. 2025 Oct 21;11:473. doi: 10.1038/s41420-025-02754-9 (PMC12540828; doi:10.1038/s41420-025-02754-9)
Supplement: Supplementary file 1 — Supplementary Figure [file 41420_2025_2754_MOESM1_ESM.docx]

**Supplementary Figure**


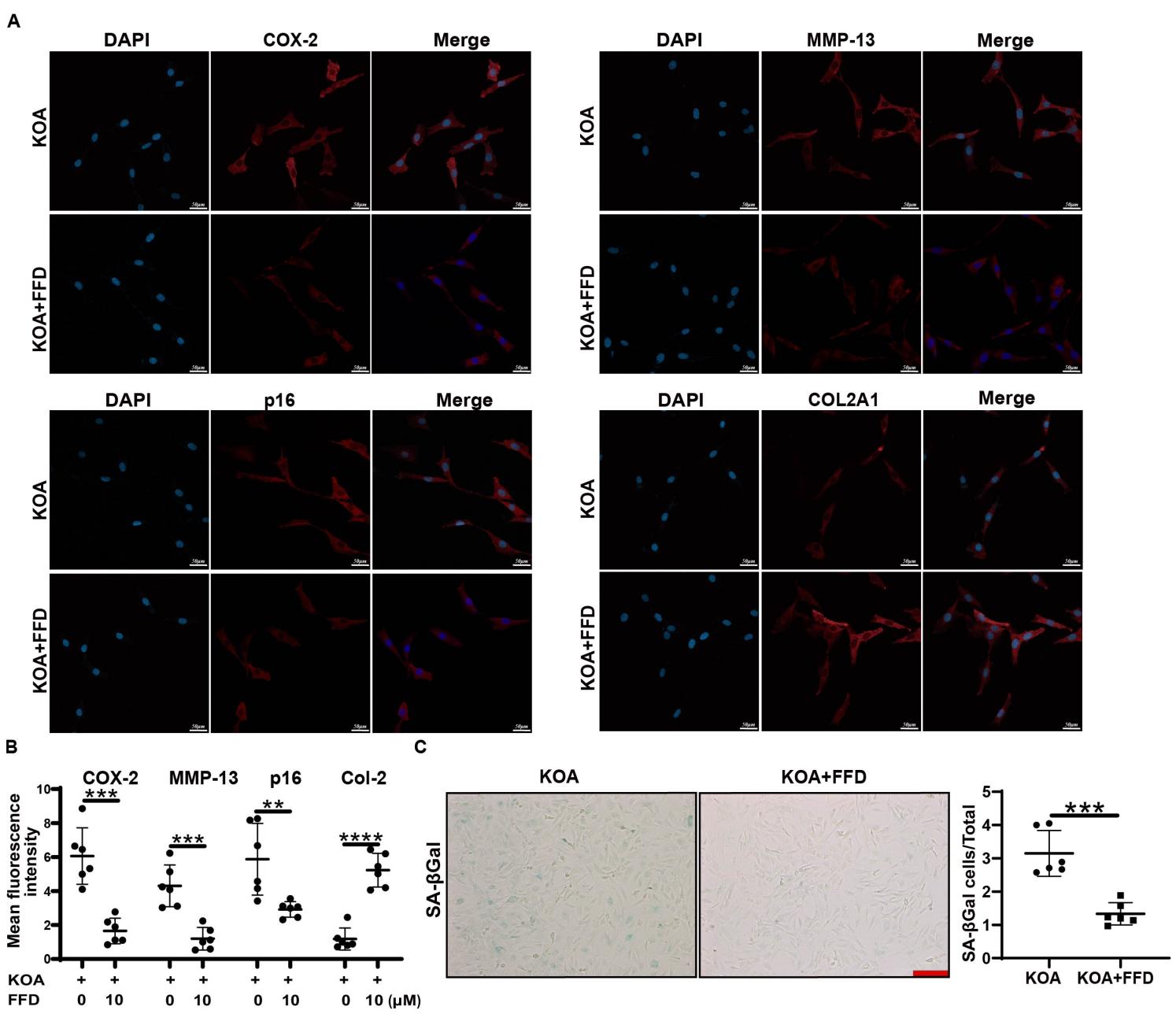


**Supplementary Figure 1. FFD regulates chondrocyte metabolism in OA ex-vivo. (A)** Chondrocytes were isolated from patients with osteoarthritis (OA) and treated with FFD (10 µM) for 48 hours, followed by immunofluorescence staining for COX-2, MMP-13, COL2A1, and p16. Scale bar=50 µm. **(B)** Quantification of immunofluorescence results was performed using ImageJ (n=6). **(C)** Representative images and quantification of SA-βGal staining in primary chondrocytes treated with FFD for 24 hours. Scale bar=50 µm, n=6 per group. Significant differences are indicated as follows: **P<0.01,***P<0.001,and ****P<0.0001.

**
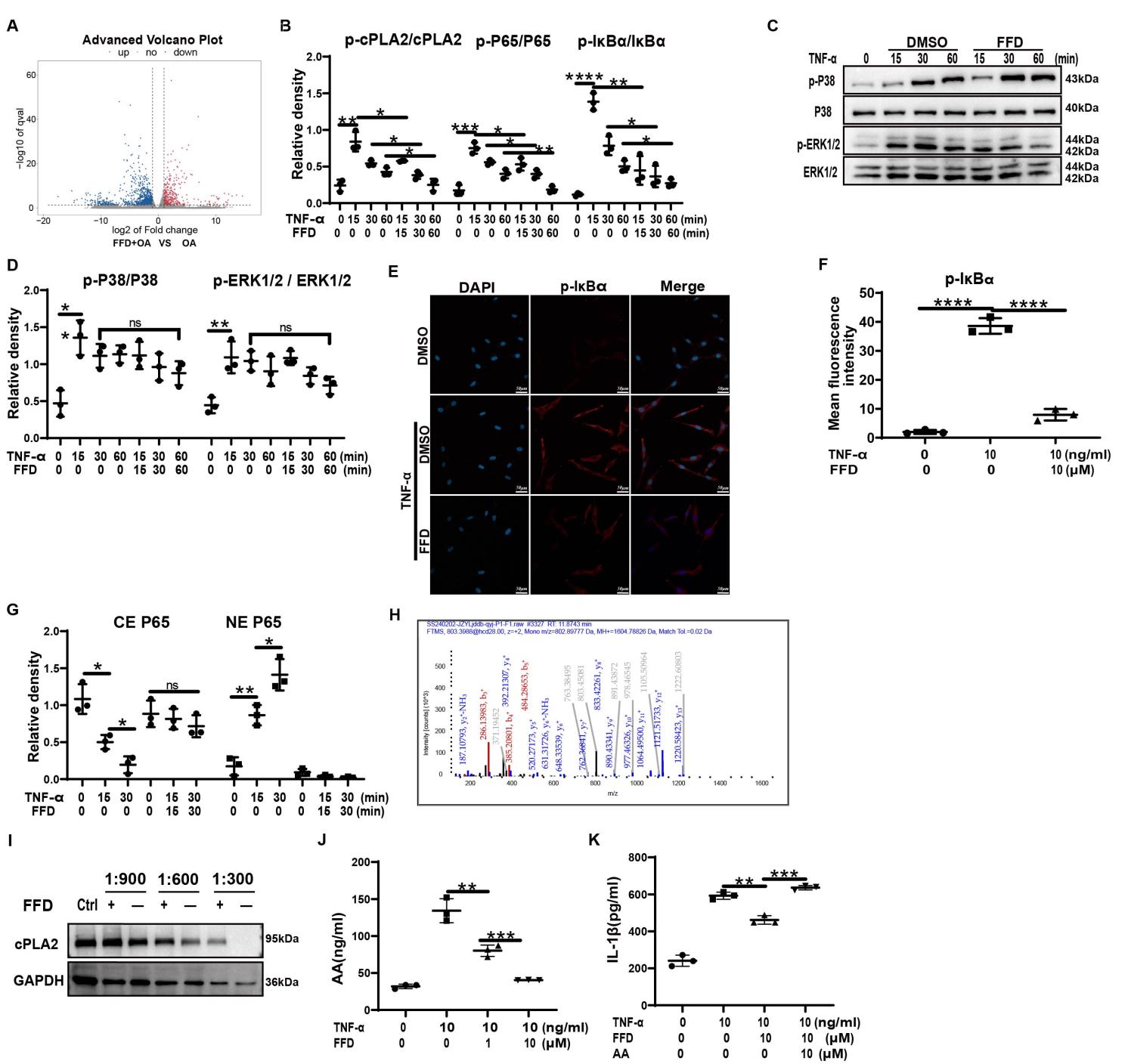
**

**Supplementary Figure 2. FFD targets the cPLA2/NF-κB signaling pathway. (A)**Volcano plot showing dysregulated genes in FFD-treated chondrocytes from patients with OA, relative to the untreated group. **(B)** Quantitative analyses of Fig.4F by Image J (n=3).  **(C-D)** The protein expression levels of the phosphorylation of p38 and ERK1/2 were detected by Western blotting. Quantitative analyses of bands by Image J (n=3). **(E-F)** Representative images and quantification of immunofluorescence staining of p-IκBα in normal human chondrocytes are shown in groups after the different treatments. Scale bar=50 µm, n=3. **(G)** Quantitative analyses of Fig.4G by Image J (n=3). **(H)** Adapted image from DARTS-mass spectrometry for cPLA2. **(I)** DARTS followed by Western blotting was used to confirm the protection of cPLA2 protein by FFD. **(J-K)** Levels of AA and IL-1β in the culture supernatants of normal human chondrocytes after different treatments were measured by ELISA (n=3). Significant differences are indicated as follows: *P<0.05 ,**P<0.01 , ***P<0.001, and ****P<0.0001; ns: not statistically significant.


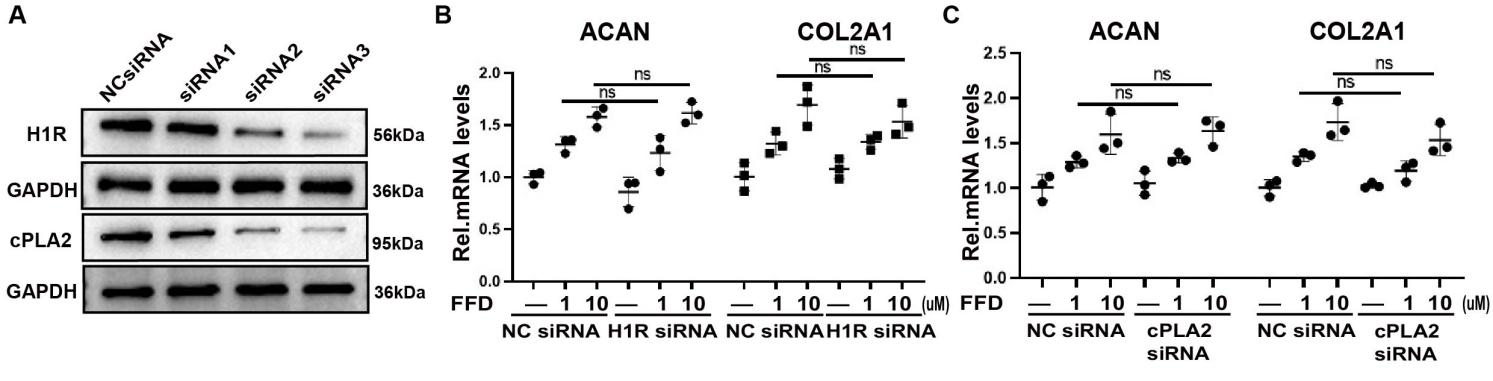


**Supplementary Figure 3. The anabolism activity of FFD is not dependent on existing targets H1R and cPLA2. (A)** Western blot analysis to examine the knockdown efficacy of siRNA against H1R and cPLA2 in the normal human chondrocyte. **(B-C)** Normal human chondrocyte transfected with different SiRNA and cultured for 24 hours with or without FFD(1, 10 µM). The mRNA levels of ACAN and COL2A1 in the chondrocyte were detected by qRT-PCR(n=3). Significant differences are indicated as follows: ns: not statistically significant.

**
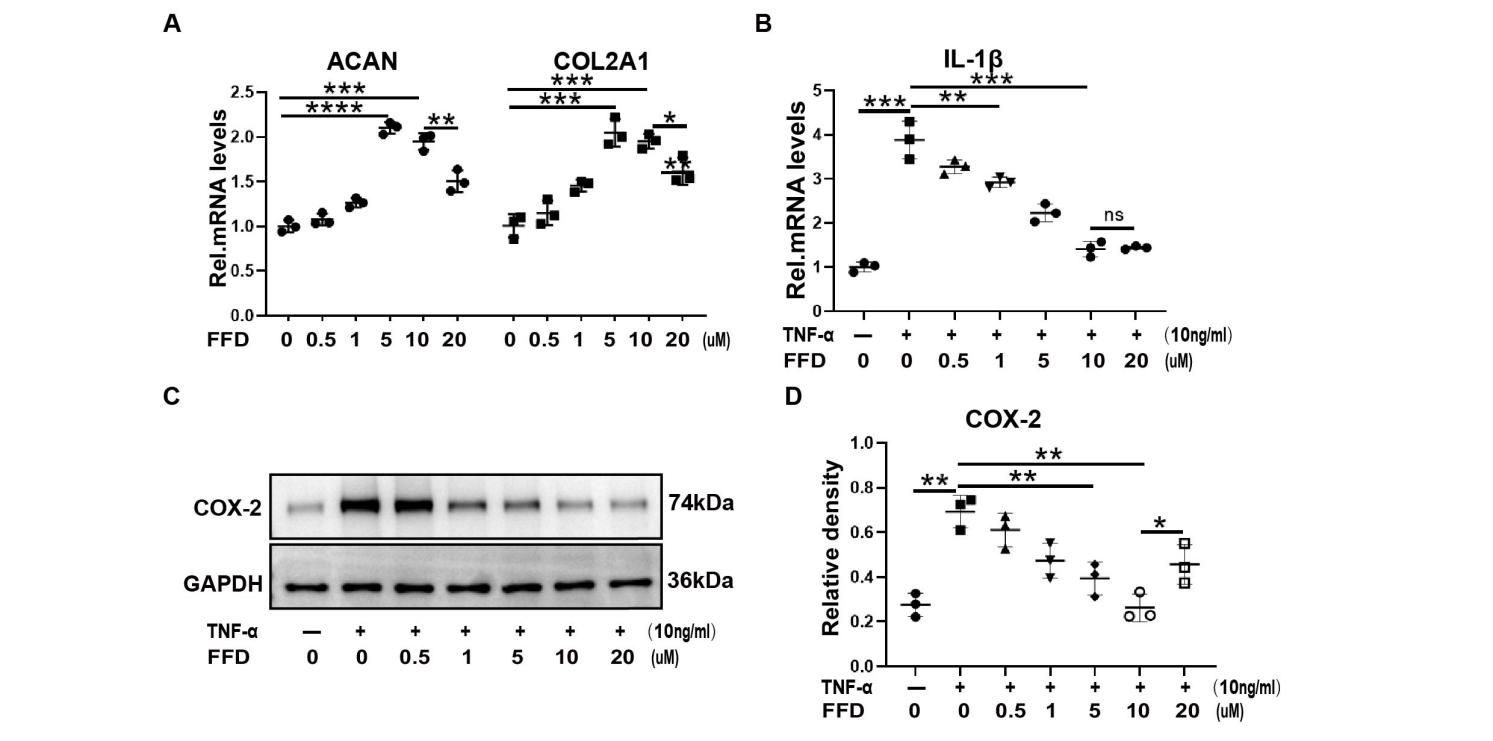
**

**Supplementary Figure 4. Selection of FFD treatment concentration. (A)** Normal human chondrocyte were treated with various FFD concentrations(0.5µM,1µM,5µM,10µM and 20µM) for 24 hours. The mRNA expression of ACAN and COL2A1 were tested by Real-time PCR. **(B)** Normal human chondrocyte were treated with TNF-α and various FFD concentrations (0.5µM,1µM,5µM,10µM and 20µM) for 24 hours.The mRNA expression of IL-1β were tested by Real-time PCR. **(C-D)** Normal human chondrocyte were treated with TNF-α and various FFD concentrations (0.5µM,1µM,5µM,10µM and 20µM) for 48 hours. The proteins expression levels of COX-2 in the different treatment groups were determined by Western blot. The intensity of the band was quantified by Image J (n=3). **(E)**Schematic depicting a proposed model for the role of FFD in osteoarthritis. Significant differences are indicated as follows: *P<0.05 ,**P<0.01 , ***P<0.001, and ****P<0.0001; ns: not statistically significant.
