# Supplementary material for: Fexofenadine protects against osteoarthritis by targeting Smad2 and STAT1 to enhance anabolism and binding cPLA2 to inhibit catabolism
Source: Cell Death Discov. 2025 Oct 21;11:473. doi: 10.1038/s41420-025-02754-9 (PMC12540828; doi:10.1038/s41420-025-02754-9)

Original Western Blots

Images of the original western blots of Fig. 1

Figure 1D. Original western blot.

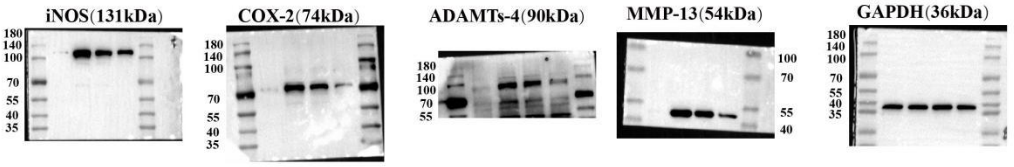

Figure 1L. Original western blot.

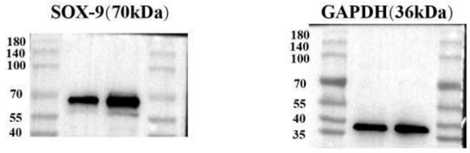

Images of the original western blots of Supplementary Fig. 2

Figure 2B. Original western blot.

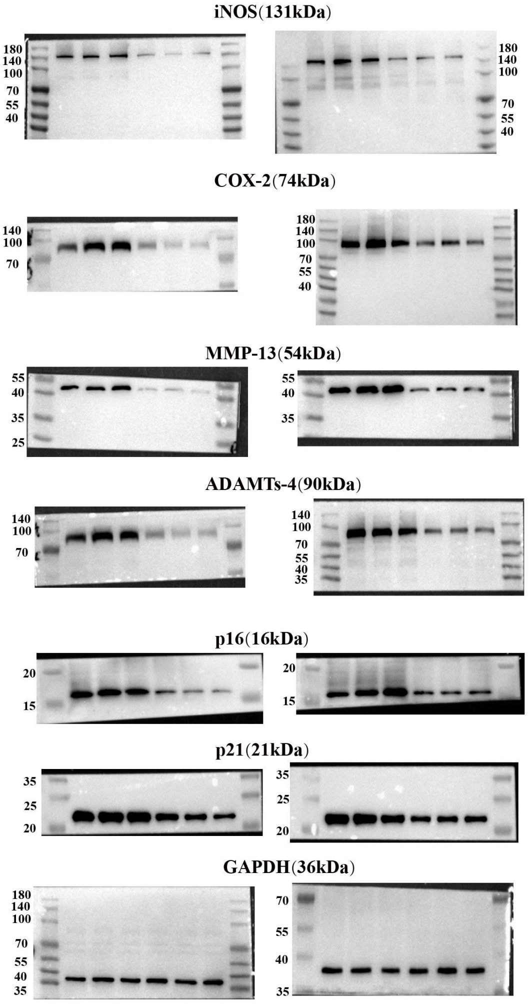

Images of the original western blots of Fig. 3

Figure 3I. Original western blot.

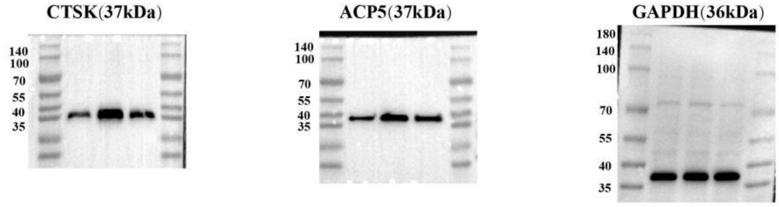

Images of the original western blots of 4

Figure 4F. Original western blot.

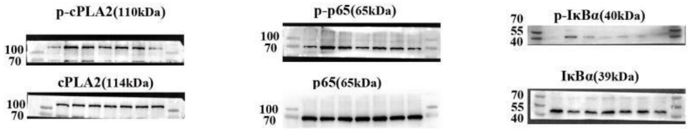

Figure 4G. Original western blot.

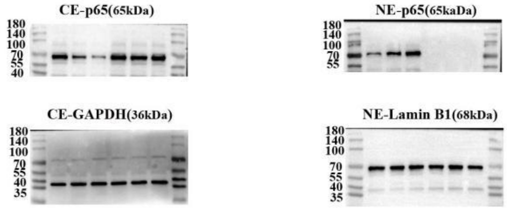

Figure 4I. Original western blot.

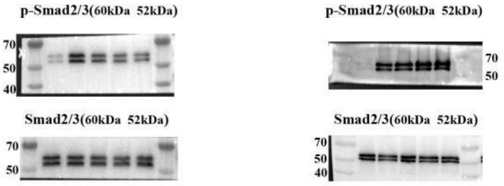

Figure 4K. Original western blot.

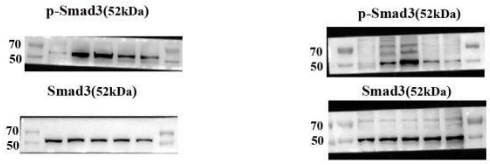

Figure 4M-N. Original western blot.

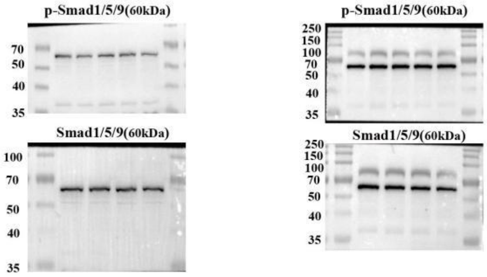

Images of the original western blots of Fig. 5

Figure 5B. Original western blot.

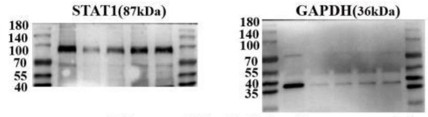

Figure 5C. Original western blot.

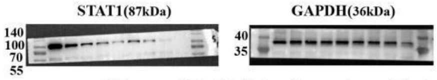

Figure 5D. Original western blot.

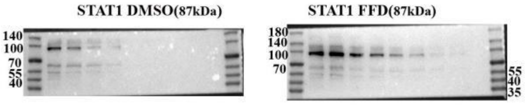

Figure 5E. Original western blot.

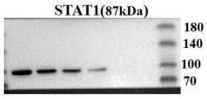

Figure 5J. Original western blot.

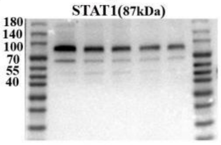

Figure 5K. Original western blot.

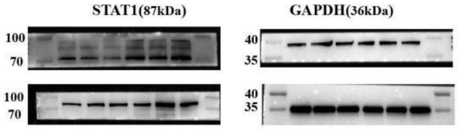

Figure 5O. Original western blot.

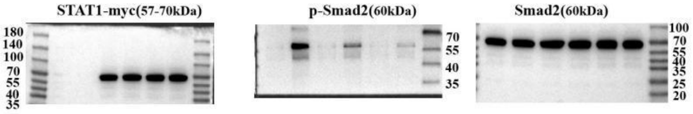

Figure 5R. Original western blot.

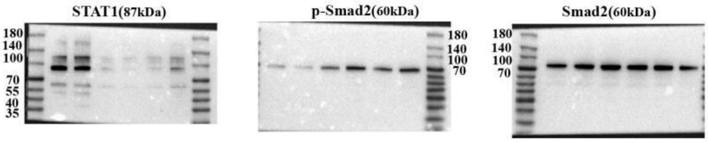

Figure 5T. Original western blot.

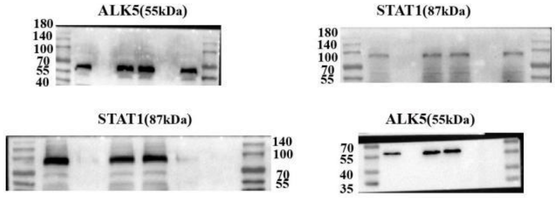

Images of the original western blots of Fig. 6

Figure 6A. Original western blot.

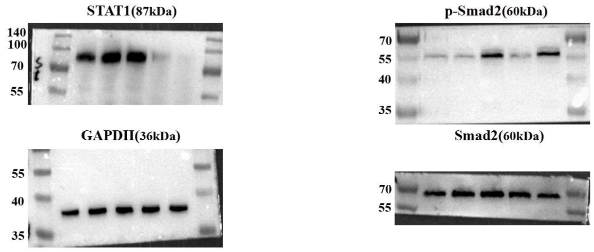

Figure 6E. Original western blot.

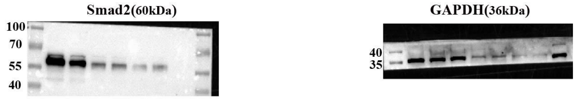

Figure 6F. Original western blot.

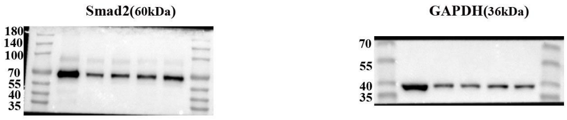

Figure 6G. Original western blot.

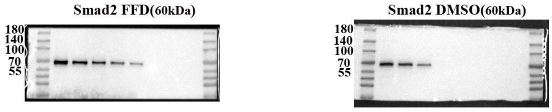

Figure 6I. Original western blot.

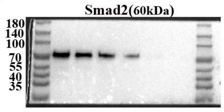

Figure 6M. Original western blot.

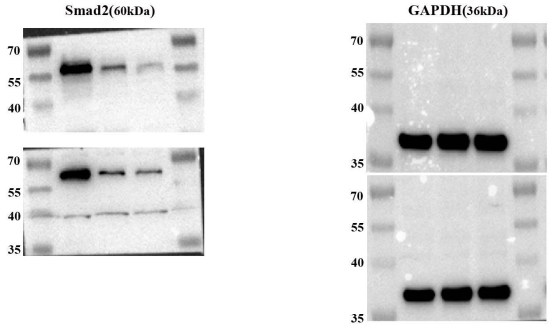

Figure 6Q. Original western blot.

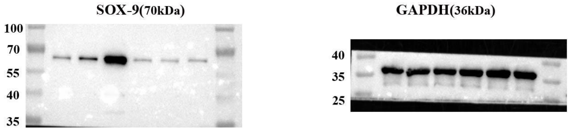

Images of the original western blots of Supplementary Fig. 2

Figure S2C. Original western blot.

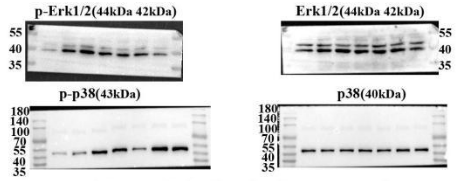

Figure S2I. Original western blot.

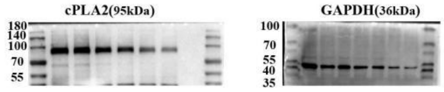

Images of the original western blots of Supplementary Fig. 3

Figure S3A. Original western blot.

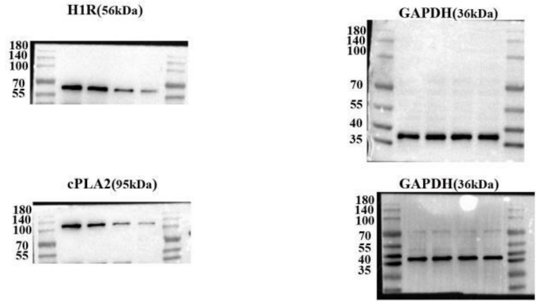

Images of the original western blots of Supplementary Fig. 4

Figure S4C. Original western blot.

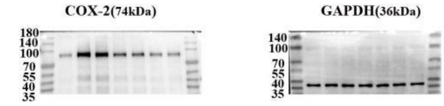

Supplement: Supplementary file 2 — Original Western Blots [file 41420_2025_2754_MOESM2_ESM.pdf]
