# Supplementary material for: Fexofenadine protects against osteoarthritis by targeting Smad2 and STAT1 to enhance anabolism and binding cPLA2 to inhibit catabolism
Source: Cell Death Discov. 2025 Oct 21;11:473. doi: 10.1038/s41420-025-02754-9 (PMC12540828; doi:10.1038/s41420-025-02754-9)
Supplement: Supplementary file 3 — Supplementary Table. 1 [file 41420_2025_2754_MOESM3_ESM.docx]

**Supplementary Table.1 Primers used for quantitative real-time PCR**

| **Target (Human)** | **Forward primers** | **Reverse primers** |
| --- | --- | --- |
| COX2 | TCAGCCATACAGCAAATCCTTG | GTCCGGGTACAATCGCGACTT |
| iNOS | CGTGGAGACGGGAAAGAAGT | GACCCCAGGCAAGATTTGGA |
| MMP13 | ATTAAGGAGCATGGCGACTTCT | GCCCAGGAGGAAAAGCATGA |
| ADAMTs4 | ATGGCTATGGGCACTGTCTC | CTGGCGGTCAGCATCATAGT |
| ACAN | GGGAAGGCTGCTATGGAGAC | ACCTCACCCTCCATCTCCTC |
| COL2 | CTGGAAAAGCTGGTGAAAGG | GGCCTGGATAACCTCTGTGA |
| COMP | CTGCAGGACTGGCTCAAAGG | CATGGTCAGGATGTATGGTGGT |
| p16 | CGCAGGTTCTTGGTCACTGT | TGTTCACGAAAGCCAGAGCG |
| p21 | CCTGGTGATGTCCGACCTG | CCATGAGCGCATCGCAATC |
| GAPDH | ACGGGAAGCTTGTCATCAAT | TGGACTCCTCGACGTACTCA |

| **Target (Mouse)** | **Forward primers** | **Reverse primers** |
| --- | --- | --- |
| ACAN | CCTGCTACTTCATCGACCCC | AGATGCTGTTGACTCGAACCT |
| COL2 | CACACTGGTAAGTGGGGCAAGACCG | GGATTGTGTTGTTTCAGGGTTCGGG |
| SOX9 | GACGTCGAAGGTCTCAATGT | ATGCTATCTTCAAGGCGCTG |
| GAPDH | CCCAGAACATCATCCCTGCATC | TCTTGATGTCATCATACTTGGCAG |
