# Supplementary material for: Fexofenadine protects against osteoarthritis by targeting Smad2 and STAT1 to enhance anabolism and binding cPLA2 to inhibit catabolism
Source: Cell Death Discov. 2025 Oct 21;11:473. doi: 10.1038/s41420-025-02754-9 (PMC12540828; doi:10.1038/s41420-025-02754-9)
Supplement: Supplementary file 4 — Supplementary Table. 2 [file 41420_2025_2754_MOESM4_ESM.docx]

**Supplementary table 2. Antibody information.**

| **Antibody** | **Company** | **Catalog #** | **Application/Dilution** |
| --- | --- | --- | --- |
| Collagen II | Abclonal | A19308 | WB (1:1000); IHC (1:100) |
| Aggrecan | Abclonal | A11691 | WB (1:1000); IHC (1:100) |
| SOX9 | Abclonal | A19710 | WB (1:1000) |
| p16 | Cell Signaling | 18769；68410 | WB (1:1000); IHC (1:100) |
| p21 | Cell Signaling | 2946 | WB (1:1000); IHC (1:100) |
| ADAMTS4 | Proteintech | 11865-1-AP | WB (1:1000) |
| MMP13 | Proteintech | 18165-1-AP | WB (1:1000); IHC (1:100) |
| COX2 | Proteintech | 66351-1-Ig | WB (1:1000); IHC (1:100) |
| iNOS | Proteintech | 22226-1-AP | WB (1:1000) |
| cPLA2 | Immunoway | YT5176 | WB (1:1000) |
| p-cPLA2 | Immunoway | YP0868 | WB (1:1000) |
| cPLA2 | Cell Signaling | 2832 | WB (1:1000) |
| IκBα | Cell Signaling | 4812S | WB (1:1000) |
| p-IκBα | Cell Signaling | 2859S | WB (1:1000) |
| p-p65 | Cell Signaling | 3033S | WB (1:1000) |
| p65 | Cell Signaling | 8242S | WB (1:1000); IF (1:100) |
| p38 | Cell Signaling | 8690 | WB (1:1000) |
| p-p38 | Cell Signaling | 4511 | WB (1:1000) |
| Erk1/2 | Cell Signaling | 4695 | WB (1:1000) |
| p-Erk1/2 | Cell Signaling | 8544 | WB (1:1000) |
| Smad2 | Cell Signaling | 5339 | WB (1:1000) |
| p-Smad2 | Cell Signaling | 18338 | WB (1:1000) |
| Smad3 | Cell Signaling | 9523 | WB (1:1000) |
| p-Smad3 | Cell Signaling | 9520 | WB (1:1000) |
| Smad2/3 | Cell Signaling | 8685 | WB (1:1000) |
| p-Smad2/3 | Cell Signaling | 8828 | WB (1:1000) |
| Smad1/5/9 | Abclonal | A23208 | WB (1:1000) |
| p-Smad1/5/9 | Cell Signaling | 13820 | WB (1:1000) |
| STAT1 | Cell Signaling | 14994 | WB (1:1000) |
| ALK5 | Abcam | ab235578 | WB (1:1000);IP(1:30) |
| H1R | Abclonal | A1422 | WB (1:1000) |
| c-Myc | Affinity | AF6054 | WB (1:1000) |
| lamin B1 | Abcam | ab16048 | WB (1:1000) |
| CTSK | Cell Signaling | 57056 | WB (1:1000) |
| ACP5 | Abclonal | A2528 | WB (1:1000) |
| GAPDH | Affinity | AF7021 | WB (1:10000) |
